# SPLF/SMFU/SRLF/SFAR/SFCTCV Guidelines for the management of patients with primary spontaneous pneumothorax

**DOI:** 10.1186/s13613-023-01181-2

**Published:** 2023-09-19

**Authors:** Stéphane Jouneau, Jean-Damien Ricard, Agathe Seguin-Givelet, Naïke Bigé, Damien Contou, Thibaut Desmettre, Delphine Hugenschmitt, Sabrina Kepka, Karinne Le Gloan, Bernard Maitre, Gilles Mangiapan, Sylvain Marchand-Adam, Alessio Mariolo, Tania Marx, Jonathan Messika, Elise Noël-Savina, Mathieu Oberlin, Ludovic Palmier, Morgan Perruez, Claire Pichereau, Nicolas Roche, Marc Garnier, Mikaël Martinez

**Affiliations:** 1 Service de Pneumologie, Centre de Compétences pour les Maladies Pulmonaires Rares, IRSET UMR 1085, Université de Rennes 1, Hôpital Pontchaillou, 2 rue Henri Le Guilloux, Rennes Cedex 9, 35033 Rennes, France; 2 Université Paris Cité, AP-HP, DMU ESPRIT, Service de Médecine Intensive Réanimation, Hôpital Louis Mourier, 178 Rue des Renouillers, 92700 Colombes, INSERM IAME U1137, Paris, France; 3 Département de Chirurgie, Institut du Thorax Curie-Montsouris, Institut Mutualiste Montsouris, et Université Paris Sorbonne Cite, 42 Bd Jourdan, 75014 Paris, France; 4 Département Interdisciplinaire d’Organisation du Parcours Patient, Médecine Intensive Réanimation, Gustave Roussy, 114 Rue Edouard Vaillant, 94805 Villejuif, France; 5 Réanimation Polyvalente, Centre Hospitalier Victor Dupouy, 69, rue du Lieutenant-colonel Prudhon, 95107 Argenteuil, France; 6 Emergency Department, Laboratory Chrono-environnement, UMR 6249 Centre National de La Recherche Scientifique, CHU Besançon, Université Bourgogne Franche-Comté, 3 Bd Alexandre Fleming, 25000 Besançon, France; 7 Samu-Smur 69, CHU Edouard-Herriot, Hospices Civils de Lyon, 5 Pl. d’Arsonval, 69003 Lyon, France; 8 Emergency Department, Hôpitaux Universitaires de Strasbourg, Icube UMR 7357, 1 Place de l’hôpital, BP 426, 67091 Strasbourg, France; 9 Emergency Department, Centre Hospitalier Universitaire de Nantes, 5 All. de l’Ile Gloriette, 44000 Nantes, France; 10 Service de Pneumologie, Centre Hospitalier Intercommunal de Créteil, Unité de Pneumologie, GH Mondor, IMRB U 955, Equipe 8, Université Paris Est Créteil, 40 Av. de Verdun, 94000 Créteil, France; 11 Service de Pneumologie, G-ECHO: Groupe ECHOgraphie Thoracique, Unité de Pneumologie Interventionnelle, Centre Hospitalier Intercommunal de Créteil, 40 Av. de Verdun, 94000 Créteil, France; 12 CHRU de Tours, Service de Pneumologie et Explorations Respiratoires Fonctionnelles, 2, boulevard tonnellé, 37000 Tours, France; 13 Département de Chirurgie, Institut du Thorax Curie-Montsouris, Institut Mutualiste Montsouris, 42 Bd Jourdan, 75014 Paris, France; 14 Université Paris Cité, Inserm, Physiopathologie et Épidémiologie des Maladies Respiratoires, Service de Pneumologie B et Transplantation Pulmonaire, AP-HP, Hôpital Bichat, 46 Rue Henri Huchard, 75018 Paris, France; 15 Service de Pneumologie et soins Intensifs Respiratoires, G-ECHO: Groupe ECHOgraphie Thoracique, CHU Toulouse, 24 Chemin De Pouvourville, 31059 Toulouse, France; 16 Emergency Department, Hôpitaux Universitaires de Strasbourg, 1 Place de l’hôpital, BP 426, 67091 Strasbourg, France; 17 Pôle Anesthésie Réanimation Douleur Urgences, Nîmes University Hospital, 4 Rue du Professeur Robert Debré, 30900 Nîmes, France; 18 Emergency department, Hôpital Européen Georges Pompidou, 20 Rue Leblanc, 75015 Paris, France; 19 Médecine Intensive Réanimation, Centre Hospitalier Intercommunal de Poissy Saint Germain, 10 Rue du Champ Gaillard, 78300 Poissy, France; 20 Service de Pneumologie, Hôpital Cochin, APHP Centre Université Paris Cité, UMR1016, Institut Cochin, 27 Rue du Faubourg Saint-Jacques, 75014 Paris, France; 21 Sorbonne Université, AP-HP, GRC29, DMU DREAM, Service d’anesthésie-Réanimation et Médecine Périoperatoire Rive Droite, site Tenon, 4 Rue de la Chine, 75020 Paris, France; 22 Pôle Urgences, Centre Hospitalier du Forez, & Groupement de Coopération Sanitaire Urgences-ARA, Av. des Monts du Soir, 42600 Montbrison, France

**Keywords:** Pneumothorax, Pleura, Outpatient, Minimally invasive, Chest tube

## Abstract

**Introduction:**

Primary spontaneous pneumothorax (PSP) is the presence of air in the pleural space, occurring in the absence of trauma and known lung disease. Standardized expert guidelines on PSP are needed due to the variety of diagnostic methods, therapeutic strategies and medical and surgical disciplines involved in its management.

**Methods:**

Literature review, analysis of the literature according to the GRADE (Grading of Recommendation, Assessment, Development and Evaluation) methodology; proposals for guidelines rated by experts, patients and organizers to reach a consensus. Only expert opinions with strong agreement were selected.

**Results:**

A large PSP is defined as presence of a visible rim along the entire axillary line between the lung margin and the chest wall and ≥ 2 cm at the hilum level on frontal chest X-ray. The therapeutic strategy depends on the clinical presentation: emergency needle aspiration for tension PSP; in the absence of signs of severity: conservative management (small PSP), needle aspiration or chest tube drainage (large PSP). Outpatient treatment is possible if a dedicated outpatient care system is previously organized. Indications, surgical procedures and perioperative analgesia are detailed. Associated measures, including smoking cessation, are described.

**Conclusion:**

These guidelines are a step towards PSP treatment and follow-up strategy optimization in France.

**Supplementary Information:**

The online version contains supplementary material available at 10.1186/s13613-023-01181-2.

## Introduction

Primary spontaneous pneumothorax (PSP) is a common disease, with a therapeutic strategy depending on its size and tolerance [1–6]. This strategy needs better codification [7–13] taking into account the risks of recurrence [14] and the expertise of the treating center.

These clinical practice guidelines were developed jointly by the French Speaking Society of Respiratory Diseases (SPLF), the French Society of Emergency Medicine (SFMU), the French Intensive Care Society (SRLF), the French Society of Anesthesia & Intensive Care Medicine (SFAR) and the French Society of Thoracic and Cardiovascular Surgery (SFCTCV) We focused on PSP of adult patients (>15 years-old).

## Method

A panel of experts from these five scientific societies, involved in the management of PSP, were gathered. The items to be addressed were defined, and formulated in "Patients/Population, Intervention, Comparison, Outcomes (PICO)” format. A literature review of French or English language, published from 2005 (and 1990 for randomized controlled trials (RCTs)) *via* the PubMed database was conducted, and performed according to the Grading of Recommendations assessment, Development and Evaluation (GRADE) methodology. The working group issued "strong" (the group recommends to/recommends not to...) or "conditional" (the group suggests to/suggests not to...) guidelines, or an "expert opinion" ((the group proposes to/proposes not to) when the level of evidence in the literature was lacking. They were then assessed by the experts and by 2 patients, and rated using a scale ranging from 1 (complete disagreement) to 9 (complete agreement). To validate a guideline on a criterion, at least 50% of experts had to express an agreement and less than 20% a disagreement. To consider an agreement as strong, at least 70% of participants had to express an agreement. In the absence of strong agreement, the guidelines were reworded until a consensus was reached. Only expert opinions that obtained a strong agreement were included. A strong agreement was reached for all the guidelines in this document.

## Summary of the results

### Definition of PSP

A spontaneous pneumothorax (SP) is defined as the presence of air in the pleural space, occurring in a non-traumatic context [2, 4, 15]. PSP is a SP in a patient without any known underlying lung disease.

### Diagnosis of PSP and initial assessment

**Fig. 1:**
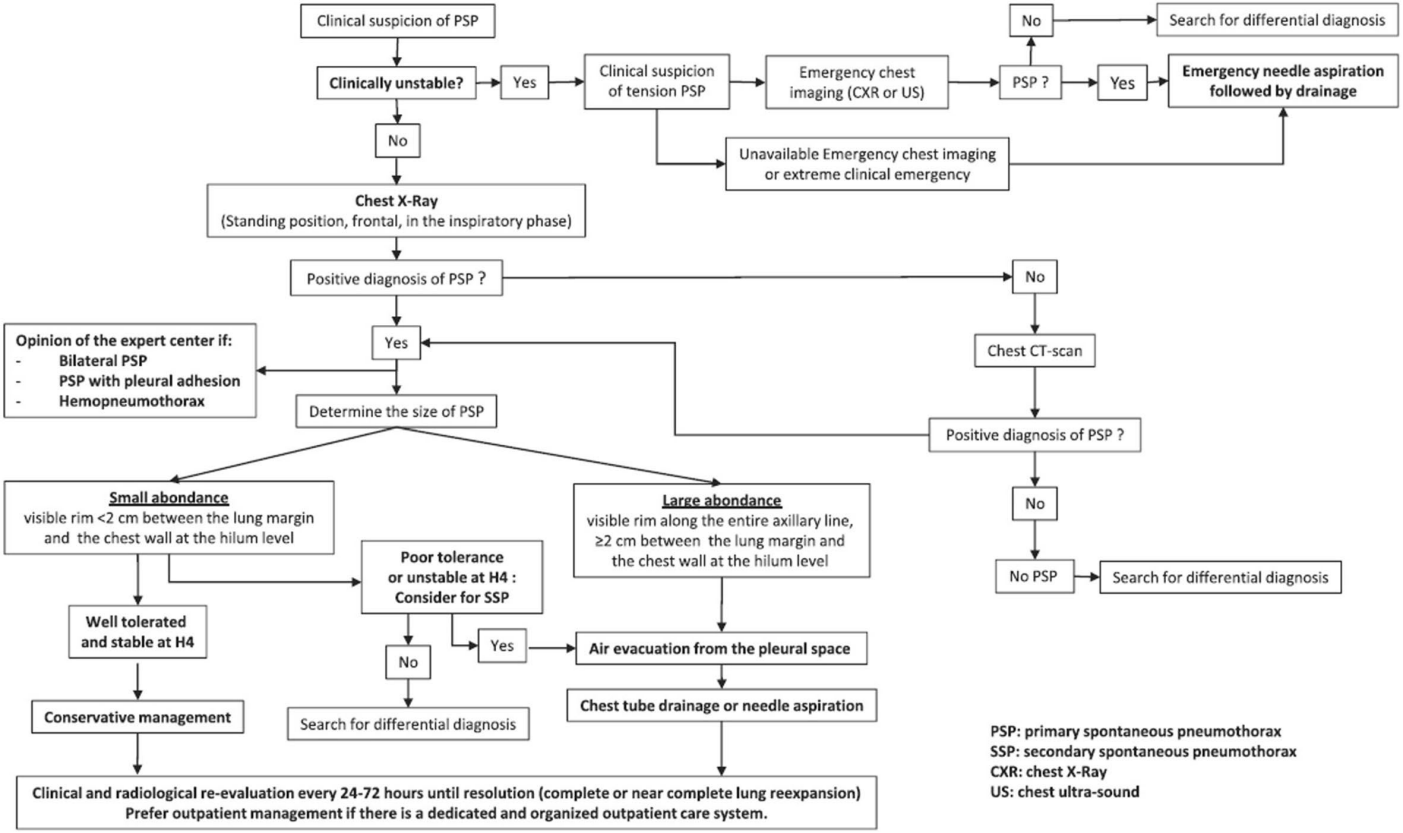
Algorithm for PSP management

The diagnostic methods are summarized in the algorithm (Fig. 1).

### Size of the pneumothorax

Defining the size of a PSP is challenging and implies a different therapeutic management among small and large PSP. The definitions of large pneumothorax vary depending on the different scientific societies and publications (Additional file 1: Appendix S2) [1–4, 6, 16].

**The group suggests to consider a PSP as large when there is a visible rim along the entire axillary line, ≥ 2 cm between the lung margin and the chest wall at the hilum level. (Conditional recommendation, low level of evidence)**

### Diagnostic imaging

#### Is chest CT-scan superior to chest X-ray (CXR) for the diagnosis, to determine the size of a PSP or to for its differential diagnosis?

The chest CT-scan is an alternative to CXR for the diagnosis of pneumothorax in the absence of signs of severity, in case of diagnostic doubt [17, 18].

The chest CT-scan is superior to the CXR for assessing the size of a pneumothorax and to determine secondary pneumothorax etiologies [19]. However, the cost, time and radiation exposure do not support its use as a first-line examination.

**The group recommends to perform a low-dose chest CT-scan in case of persistent diagnostic doubt despite the investigations already performed. (Strong recommendation, low level of evidence)**

**The group proposes that, although the chest CT-scan is superior to CXR for the positive diagnosis of PSP, assessing its size and ruling out a differential diagnosis, its cost, radiation exposure and accessibility do not support its use as a first-line examination. (Expert opinion)**

**The group proposes to perform frontal CXR in inspiration, without expiratory films, in case of suspected PSP to diagnose it and assess its size. (Expert opinion)**

#### Is chest ultrasound superior to CXR for the positive diagnosis and to determine size of PSP?

The added value of chest ultrasound is currently for chest trauma patients or the diagnosis of iatrogenic pneumothorax, with a high pretest probability [20–29].

Routine chest ultrasound for diagnosis and size assessment of PSP without severity signs is not defined. However, its high sensitivity access easy in case of clinical emergency, chest ultrasound may an alternative in experienced teams.

**The group suggests not to solely base the diagnosis of PSP on chest ultrasound in the absence of signs of severity. (Conditional recommendation, low level of evidence)**

The literature does not allow concluding on the place of chest ultrasound as an alternative to CXR [30–32].

**The group proposes not to solely rely on chest ultrasound to assess the size of a PSP. (Expert opinion)**

**No data in the literature allow concluding on the value of chest ultrasound to rule out the differential diagnoses of PSP.**

#### Is chest ultrasound superior to CXR for follow-up after drainage?

No standardized guideline on the frequency and number of CXR for the follow-up of a pneumothorax exists.

Drainage is pursued until complete lung expansion and after bubbling has stopped [33]. Two studies compared CXR and ultrasound for the follow up of patients drained for a PSP [34, 35]: ultrasound was superior to CXR for the follow-up, and the level of experience of the operators was equivalent, provided a 2 h specific training.

**The group suggests performing chest ultrasound for the diagnosis of residual pneumothorax in patients drained for pneumothorax. In untrained teams or teams with limited access to ultrasound, CXR may be used as an alternative. (Conditional recommendation, moderate level of evidence)**

### Therapeutic management of PSP

The therapeutic methods are summarized in the algorithm (Fig. 1).

### Patient with sign(s) of immediate severity

#### Clinical definition

Clinical definition. Respiratory distress or haemodynamic instability in PSP is rare [36, 37]. In well-tolerated PSP, therapeutic strategy can be discussed according to location, size, first episode or recurrence, complication and patient’s characteristics [38, 39].

**The group recommends to consider that tension pneumothorax is defined by respiratory distress or haemodynamic instability. (Strong recommendation, low level of evidence)**

#### Extreme emergency = chest decompression

Tension pneumothorax is a gas tamponade [37]. Bedside emergency chest decompression after chest imaging can reverse this life-threatening condition [36, 38].

**In case of confirmed tension PSP, the group recommends:**

- **Emergency chest decompression,**
- **Through an anterior (mid-clavicular line in the 2nd intercostal space) or axillary (mid-axillary line in the 4th intercostal space) approach,**
- **Using dedicated equipment (thoracentesis kit) or any other needle aspiration device available to the operator. (Strong recommendation, low level of evidence)**

### Patient without signs of immediate severity

#### Large and/or symptomatic PSP without sign of immediate severity

In international studies, large PSPs are "mixed" with symptomatic PSPs. The main symptom of PSP is dyspnea. In order to facilitate the reading, we used the term "large PSP" instead of "large or symptomatic PSP" in these guidelines.

We performed pairwise comparisons of the four management methods for patients with large PSP [2, 4, 15]:

- Conservative strategy: no intervention, "therapeutic abstention" and monitoring,
- Surgical approach,
- Needle aspiration (NA),
- Chest tube drainage (CTD).

#### Is CTD superior to conservative strategy?

A single study has assessed the conservative strategy in patients with a first episode of a large unilateral PSP [12]. This multicentre, open-label, RCT included 316 patients aged 14 − 50 years: 154 in the CTD and hospitalization (“standard”) arm and 162 in the conservative strategy arm.

The conservative management was non-inferior to the “standard” management on the primary endpoint (eight weeks radiographic resolution) [12]. The risk of ipsilateral recurrence at one year was lower in the conservative arm (8.8% vs 16.8%) [12, 39]. However, these data should be considered with caution as 1-year recurrence rate was lower than usual in both arms (29% in the meta-analysis by Walker et al. [14]). The conservative strategy allowed reducing the number of procedures (percutaneous pleural procedures, need for surgery, imaging), the hospital length of stay, the rate of adverse events and the number of days out of work [12]. The failure rate of the conservative strategy (defined as the need for CTD) ranged between 15 and 21% [12, 40].

However, some biases limit its conclusions, rending questionable and hardly generalizable these results:

- in sensitivity analyzes, if all lost to follow-up patients are considered as “failures”, the difference in success at eight weeks exceeds the non-inferiority limit and the study is negative, with conservative management less effective than CTD;
- there might be an inclusion bias, as only 316 patients out of the 2637 screened were included;
- the type of included patients seems unusual: the duration of PSP evolution was about of 40 h, the patients being barely asymptomatic (median dyspnea and pain visual analog scale scores respectively of 1 and 2 on 10-points scales).

Further studies are needed to assess the safety and better define the profile of patients who could benefit from a conservative strategy.

**The group recommends air removal from the pleural cavity in patients with large PSP without signs of immediate severity. (Strong recommendation, moderate level of evidence)**

#### Is CTD superior to NA?

NA requires a transient pleural approach, performed until bubbling stops [41–43], until aspiration is no longer possible [44–50], until a fixed volume is aspirated [46, 50, 51], or for a maximal time [41, 43, 44]. If NA is not sufficient, studies suggest either switching to CTD [42, 43, 45, 47, 52] or to attempt a second [or a third [43]] NA [41, 44, 53]], although not recommended [4, 15]. NA failure is defined as an attempted NA followed by a CTD [41–45, 47, 52, 53].

CTD shows a higher "immediate success" rate compared to NA [41, 49, 50, 54–56]. A later success rate (at day 7 or later), appears similar between CTD and NA [44, 48, 49, 51, 55]. The overall immediate success rate is 51% for NA (after 1 − 3 procedures) [41–44, 46, 48–57] and about 68% for CTD [41, 44, 48–51, 53–57].

NA reduces the hospital length of stay compared CTD (i.e. with hospitalization) [41, 44, 46, 48–51, 54–56, 58].

NA allowed decreasing drainage duration compared to CTD in an RCT [51].

The risk of complications is rarely reported and lower during NA than CTD [44, 50, 55]. One study found similar complication rates between NA and CTD [53].

Conflicting data exist in pain. Although some report less pain [44, 55] with NA and higher analgesic use in CTD [44], older studies did not report any difference between techniques [41, 53].

Treatment satisfaction did not differ between NA and CTD [53].

Surgery rate did not differ between NA and CTD [51, 53]. A single old study, of low level of evidence, reported a more frequent need for pleurectomy in CTD as compared to NA [46].

Costs had not been analyzed in these studies. The 2017 Cochrane Database considered that no conclusion was possible [59]. However, as NA reduces the hospital length of stay as compared to CTD, the cost of such management would be lower. Dedicated medico-economic studies are needed.

One-year (and sometimes 3 months) recurrence rates did not differ between NA and CTD [41, 44, 46, 48, 49, 54, 55, 57].

Based on these findings, NA might appear superior to CTD. Given the possibility of an outpatient management with a CTD (see below), these two approaches were kept as first-line management of large PSP.

#### Is CTD superior to surgery?

Seventy percent of patients with a first PSP will never relapse. Therefore, performing surgery at first episode of PSP seems too invasive [14]. However, several studies compared surgical treatment (pleurodesis and bleb removal) following CTD or NA to a conventional CTD strategy without surgery during a first episode of large PSP. These studies reported a reduced one-year risk of recurrence, a shorter length of stay, a lower cost and no difference in analgesic for surgically treated patients compared to conventional CTD [60–65]. These studies had several biases drawing the conclusion uneasy.

**The group recommends air removal from the pleural cavity using either NA or CTD as first-line treatment in patients with large PSP without signs of immediate severity. Surgery should not be performed as first-line treatment except in specific situations (see below). (Strong recommendation, moderate level of evidence).**

#### Is outpatient superior to inpatient management?

No high-quality study has directly compared the outpatient management with NA versus mini-CTD with one-way valve. At least one such study is ongoing (PNEUM-AMBU, NCT03691480).

Exclusive outpatient management is feasible in 4 out of 5 patients treated with NA or CTD with a one-way valve [9, 10, 13].

Compared with inpatient care, outpatient management reduces the hospital length of stay, as reported in a study comparing a drainage system with an integrated one-way valve versus a standard management with NA in most cases (68% of patients in the control arm) [13]. In most other publications, outpatient NA has been compared to drainage with hospital-based monitoring [41, 44, 46, 48–50, 53–55, 57, 58]. Series reporting outpatient management have also suggested a reduced hospital length of stay but without control arm [8–10, 66].

Use of surgery was not assessed specifically in study reporting outpatient strategy.

Outpatient management could increase the complications rates as compared to inpatient management [13]. However, the design of this prospective study explains most of the severe complications, as adverse events (AEs) were defined as the need for hospitalization, which could only concern patients of the outpatient arm.

On the other hand, in observational studies [8–10, 66], no serious AEs were reported, but mainly bent or displaced catheters (ranging from 1.5% [10] to 22.6% [66]).

Outpatient management was associated with a similar 1-year recurrence rate as inpatient management [8–10, 13, 66], ranging from 12% [8] to 33.1% [9].

Indirect evidence [8, 10] suggest a lower overall health care costs of outpatient management.

**The group recommends outpatient management in patients with large PSP without signs of immediate severity. (Strong recommendation, moderate level of evidence)**

**The group recommends an outpatient management based on needle aspiration or on mini-chest tube with one-way valve, if the following criteria are met:**

- the patient is stable after intrapleural air removal,
- and a dedicated outpatient care system is previously organized
- and a consultation with chest ultrasound or CXR is scheduled at 24−72 h to assess the evolution.
- **(Strong recommendation, low level of evidence)**

**The group proposes outpatient management of PSP only if all of the following conditions are met:**

- A patient information leaflet providing guidance on the way to behave in case of problem and phone numbers available 24/7, including SAMU-Centre 15 is given to the patient before hospital discharge (examples in appendices),
- Patient’s comprehension of discharge instructions has been checked
- The patient should not stay alone for the first 24 − 48 h after being discharged home,
- The patient should be able to access a medical facility within 1 h, regardless of the means of transportation, in the event of deterioration,
- The time of discharge does not matter if all of the above criteria are met (nighttime discharge is possible). **(Expert opinion)**

### Small PSP without signs of immediate severity

The presence of signs of severity in a small PSP should prompt the clinician to consider another diagnosis such as SSP. A small PSP itself cannot cause respiratory or haemodynamic failure. Relevant signs of poor tolerance to be investigated and to guide the management are rest dyspnea or pain unresponding to non-opioid analgesics.

No study focused specifically on small PSP, only among PSP managed conservatively [8, 56].

**The group recommends conservative management for patients with small PSP without signs of poor tolerance. (Strong recommendation, low level of evidence)**

**The group recommends a conservative outpatient management for patients with small PSP without signs of poor tolerance if the following criteria are met:**

- Physical examination and CXR findings are unchanged after 4 h monitoring,
- and a dedicated outpatient care system is previously organized
- and a consultation with chest ultrasound or CXR is scheduled at 24−72 h to assess the evolution. **(Strong recommendation, low level of evidence)**

**The group proposes outpatient management of PSP only if all of the following conditions cited above are met.**

### Analgesia for medically-treated PSP

#### Analgesia during the chest procedure

No study has compared air removal from the pleural cavity using NA or CTD with and without local anesthesia. Conducting such a study nowadays seems unethical. Although the intensity of pain caused by pleural puncture, placement of a small-bore chest tube using the Seldinger technique, or placement of a large-bore CTD is not similar, these painful procedures can be completely prevented by local lidocaine anesthesia of the skin, subcutaneous tissues and intercostal muscles. As for any invasive procedure, it seems legitimate to advocate an analgesic management (at least a correctly performed local anesthesia [67]).

Most RCTs of NA and CTD have used local anesthesia [44, 48–51]. The sufficient dose of lidocaine is generally of 2 mg kg^−^^1^ (injected into the chest wall), with a maximal dose of 4–5 mg kg^−^^1^. Needle insertion should target the upper edge of the rib in order to avoid injury to the neurovascular bundle and to ensure that there is no blood reflux into the syringe through gentle aspiration before injecting.

**The group recommends performing local anaesthesia of the chest wall before air removal from the pleural cavity through NA or CTD. (Strong recommendation, low level of evidence).**

#### Analgesia

No study investigated pain management in PSP treated conservatively, with NA or with CTD. Only one RCT [68] reported a transient and partial efficacy of intrapleural injection of 20 mL of 0.5% bupivacaine every 8 h to reduce pain related to the CTD, within 60 min after the injection, but not at 4 or 8 h, and no effect on morphine use. An RCT in patients who underwent thoracic surgery reported the decrease in pain related to cough or mobilization following 20 min application of ice on chest tube insertion site [69]. No study performed in patients drained for a PSP allows a conclusion.

**The group recommends to base pain management on multimodal analgesia in patients medically treated for PSP (NA, CTD, conservative management). (Strong recommendation, low level of evidence)**

#### Analgesia during chest tube removal

Chest tube removal is a most painful procedure [70]. Analgesic management during chest tube removal is justified.

RCT assessing the efficacy of therapeutic interventions on tube removal-related pain were performed for larger-bore chest tubes (≥ 16 Fr) after cardiothoracic surgery.

The use of morphine or non-steroidal anti-inflammatory drug (NSAID) before removal seems equivalent [122]. The addition of local anesthesia with topical lidocaine-prilocaine or subcutaneous lidocaine may improve pain upon chest tube removal, especially in the absence of multimodal systemic analgesia [123–125].

The main non-pharmacological technique is cold application on and around the chest tube insertion site 15 − 20 min before its removal. Many studies explore this technique and a meta-analysis has shown a beneficial effect of cold application. Although this gain in pain scores may appear modest, this technique is recommended, especially for large-bore chest tubes [71–78].

**The group recommends a multimodal analgesia including cold application to reduce pain associated with large-bore chest tube removal (≥ 16 Fr). (Strong recommendation, low level of evidence).**

**The group proposes to use analgesia during small-bore chest tube removal, but further studies are needed to determine the preferred method of analgesia (Expert opinion)**

### Specific cases

#### Simultaneous (or synchronous) bilateral PSP

The occurrence of simultaneous bilateral pneumothorax has been described as clinical cases, in patients with a respiratory history, or during episodes of traumatic pneumothorax. This rare condition can also occur in case of iatrogenic or idiopathic mediastinal fenestration between the right and left pleural cavities (known as "buffalo chest") [79–86].

**In case of simultaneous bilateral PSP, regardless of its size, the group proposes to contact as soon as possible an expert centre, i.e. a centre with a thoracic surgery department, to discuss the treatment approach and to consider a transfer to this centre. (Expert opinion)**

**In case of simultaneous bilateral PSP with signs of severity or large PSP, the group proposes to perform emergency CTD. (Expert opinion)**

#### Primary spontaneous haemopneumothorax

Spontaneous haemopneumothorax (SHP) is defined as a PSP associated with the presence of a variable volume of blood in the pleural cavity. Any spontaneous pleural air-fluid level should be suspected to be a SHP. If possible, it is justified to prove the presence of a SHP by draining it. SHP accounts for 1 − 12% of PSP [87–90].

In case of SHP, CTD is indicated. According to Boersma et al., surgery is indicated in case of haemorrhagic shock, if the accumulated blood volume exceeds 1500 mL or if bleeding exceeds 200 mL h^-^^1^ for at least two hours [91]. A surgical approach is not questionable in case of haemodynamic instability, but debated in its absence [87, 89, 90, 92–95].

**In case of haemopneumothorax, regardless of its size, the group proposes to contact as soon as possible an expert centre, i.e. a centre with a thoracic surgery department, to discuss the treatment approach and to consider a possible transfer to this centre. (Expert opinion)**

**In case of haemopneumothorax with signs of severity or large haemopneumothorax, the group proposes to perform emergency CTD. (Expert opinion)**

#### PSP with pleural adhesion

Pleural adhesion is a risk factor for haemothorax when present on CXR at the time of the diagnosis of PSP. Pleural adhesion disruption may lead to a massive or fatal SHP of systemic origin [90, 96].

**In case of PSP with confirmed pleural adhesion, regardless of its size, the group proposes to contact as soon as possible an expert centre, i.e. a centre with a thoracic surgery department, to discuss the treatment approach and to consider a possible transfer to this centre. (Expert opinion)**

**In case of PSP with confirmed pleural adhesion and signs of severity or large PSP, the group proposes to perform emergency CTD. (Expert opinion)**

### Chest tube drainage

#### Is small-bore superior to large-bore chest tube?

For several years, the guidelines advocated the use of small-bore chest tubes (≤ 14 Fr) for the management of pneumothorax [4]. Their efficacy is equivalent and the complication rate is lower: 5 to 9.5% vs. 27 to 32% [44, 57, 97, 98]. Among the complications of small-bore chest tubes, obstructions and displacements occurred in 1 − 5% of cases [99].

However, when choosing the drainage approach, other elements must be taken into account, such as the technique (Seldinger vs. internal stylet tube) or the shape of the tube (straight vs. pigtail tube) [100].

**The group suggests to use a small-bore chest tube (≤ 14 Fr) for CTD of PSP. (Conditional recommendation, low level of evidence).**

#### In case of removal of air from the pleural cavity, should the axillary over the anterior approach be preferred?

In most studies, NA is performed anteriorly in the second or third intercostal space on the midclavicular line and CTD in the fourth or fifth intercostal space on the middle or anterior axillary line.

The anterior route carries more vascular risks (subclavian or internal thoracic vessels). These risks are significantly reduced with ultrasound guiding [101]. The anterior approach allows positioning the tube where the air is accumulated, but may cause visible scarring.

The axillary approach is considered to be safer, especially in the safety triangle, but there is a risk of diaphragmatic and underlying organ trauma, or risk for the axillary vessels. An ultrasound study has found the diaphragm in the safety triangle in 20% of cases, highlighting the interest of systematic ultrasound location before CTD [102]. The tube should be positioned upwards, a position achieved in less than half of the cases [97, 100].

The anterior approach has been used when assessing the outpatient management with a small-bore tube sealed with a one-way valve, [8–10, 103].

A higher risk of plication and obstruction of the NA catheter using the axillary approach compared to the anterior approach has been reported in an animal model of emergency NA of pneumothorax [104], while no difference in tube displacement between both approaches had been reported in humans. It is therefore impossible to conclude on the superiority of any approach [105].

**The literature does not provide sufficient data to choose between the anterior and axillary approach.**

**The group suggests to obtain an ultrasound visualization before performing needle aspiration or CTD using the anterior or axillary approach, in order to reduce the risk of complications. (Conditional recommendation, low level of evidence)**

#### In case of CTD, is suction superior to free flow?

The guidelines do not comment on suction via the chest tube at chest tube insertion [4, 15, 106].

Studies comparing NA to CTD do not show any difference in efficacy whether initial suction is applied or not [41, 46, 48, 49, 53].

In outpatient management studies, no suction was applied, with passive evacuation through the one-way valve, with satisfactory the success rates [4, 9, 10, 13, 15, 106].

Two RCTs studies have assessed the benefit of suction, without any difference in efficacy [107] or recurrence rates [108]. Conversely, the risk of "a vacuo" pulmonary oedema or reexpansion oedema [109] seems rare and has not been described in any of the studies comparing drainage and NA [110]. However, a rate of 16% has been reported [111], with diabetes and large pneumothorax as independent risk factors [112], while avoiding a too rapid air evacuation [112] is recommended.

In an animal model of induced pneumothorax [113] the duration of pneumothorax and suction have been identified as the two risk factors for reexpansion oedema.

**The group recommends to initiate drainage with passive air evacuation (one-way valve or free flow) and to subsequently start suction at − 5 to − 20 cm H**_**2**_ **0 only if reexpansion is not achieved. (Strong recommendation, moderate level of evidence)**

#### In case of CTD, is clamping the chest-tube before removal necessary, and does it reduce the risk of recurrence?

Several studies have assessed a clamping trial before tube removal, with conflicting results. A retrospective study found more frequent recurrences after CTD removal than in the absence of clamping trial [114], while another did not report any difference in pneumothorax recurrence [115]. Of note, in both, tension pneumothorax happened only in the clamping group [114, 115].

Two RCT have been performed in traumatic haemothorax and pneumothorax (haemopneumothorax) [33, 116]. In both, no difference was found in recurrence rate of pneumothorax with or without clamping trial [116]. However, suction strategies differed between groups, with recurrences in the clamped group needing declamping, while recurrences after removal of the drain requires to repeat the drainage procedures; moreover, the included population trends these findings poorly applicable to PSP.

**In patients under chest tube suction, in the absence of bubbling and with lung re-expansion, the group proposes to allow a free flow for 6–8 h before tube removal to avoid a new drainage procedure in case of early recurrence. (Expert opinion)**

**The data in the literature do not allow concluding on the interest of performing a clamping trial before tube removal once the lung is re-expanded.**

### Additional treatments in PSP

#### Benefit of oxygen therapy in PSP treatment

Systematic oxygen therapy has been proposed in order to increase the resolution rate of conservatively-treated PSP, based on the assumption that oxygen administration would reduce the partial pressure of nitrogen in the alveolar space compared to that in the pleural cavity, and promote the passage of nitrogen from the pleural cavity to the alveolar space via the pleural capillaries.

Some retrospective clinical studies suggest an increased resolution rate in patients treated with systematic oxygen therapy, especially in the subgroup of patients with large pneumothorax (> 30%) [117], even with low-flow oxygen therapy (2–4 L min^−1^) [118]. In neonates, studies do not show any increase in clinical recovery rate from pneumothorax with systematic administration of oxygen therapy [119, 120].

Poor methodological quality of the data, and the many potential disadvantages of the systematic administration of oxygen (need for hospitalization with additional costs, discomfort, bed rest) do not support the systematic administration of such a treatment for the management of PSP patients treated conservatively.

**The group does not recommend the systematic use of oxygen therapy in patients treated for PSP. (Strong recommendation, moderate level of evidence).**

#### Benefit of strict rest during the conservative strategy

No study compared strict bed rest with no activity limitation. In the only RCT comparing a conservative strategy with invasive treatment with CTD for the first episode of moderate-to-large PSP [12, 121], no instructions for activity limitation were given. Similarly, no guideline regarding activity were provided in the assessing the conservative strategy for recurrent SSP [122]. The ACCP, BTS and ERS task force guidelines do not recommend any activity limitation with the conservative strategy [2, 4, 15]. The sole "activity limitation" the patients should be warned is the risk of air travel in the presence of a pneumothorax, (see below) [123–125].

In the absence of evidence of a relationship between the recurrence and physical exertion, the patient may be advised to return to work and resume normal physical activities once symptoms have resolved. It seems reasonable to advise postponing sports involving extreme exertion and physical contact until complete resolution.

**The group suggests not to prescribe strict bed rest in PSP patients. (Conditional recommendation, low level of evidence)**

**The group proposes to limit intense or contact sports activities until complete resolution of the pneumothorax. (Expert opinion)**

### Special cases of medical transport

**Fig. 2:**
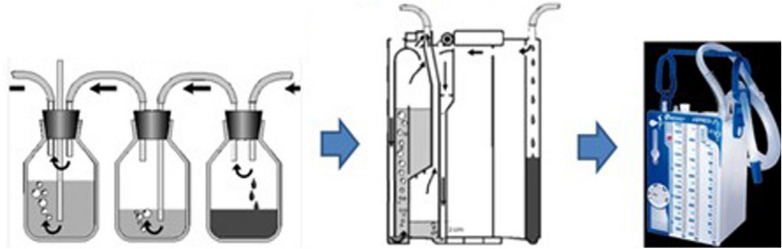
Single-use portable unit that applies the 3-bottle principle in a single device

**Fig. 3:**
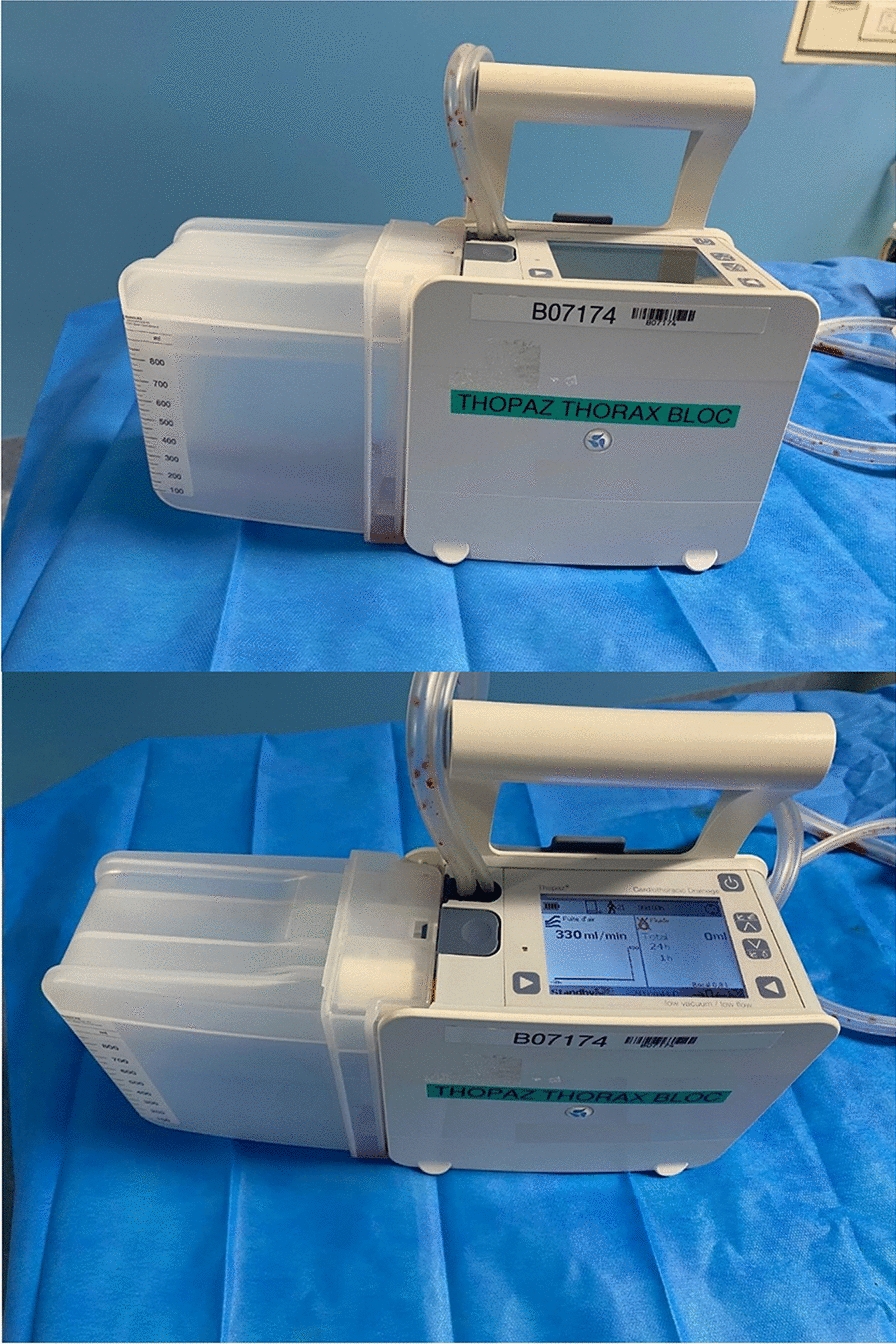
Example of a digital drainage system

Transporting a patient with a drained PSP holds some risks, and might be a source of complications. A bubbling tube should never be clamped. A tube should never be clamped in case of positive pressure ventilation due to the risk of overpressure [37, 126]. In case of CTD of a PSP, the tube, attached to a flexible transparent tubing with a connection for sealing, is connected to a collection device equipped with an anti-reflux system, always placed vertically, about 40 cm below the patient’s thorax. The Heimlich valve is a one-way anti-reflux valve, used when no valve is integrated into the collection device [127]. Closed collection systems are recommended (Fig. 2), allowing a reliable monitoring of the negative pressure applied. Portable digital suction drainage devices are available (Fig. 3). When positioning the patient for transport, care should be taken to ensure that the tube is not bent or clamped. The tube dressing and the occurrence of subcutaneous emphysema should be visually monitored.

For the transport of patients with CTD, fitting a stand-alone suction device to the compact system allows the drainage system to be used as a stand-alone suction unit [126].

In case of pneumothorax, air transport theoretically holds some risks because of the altitude [128] (see below).

**To reduce the risk of tension pneumothorax, the group proposes to organize the transportation of patients with drained SP as follows:**

- In the absence of bubbling: with a tube attached to a one-way valve,
- In the presence of bubbling: by continuing continuous suction with fitting of a stand-alone suction pump connected to the 3-compartments drainage system. (Expert opinion)

### Place of surgery

#### What are the indications for the surgical management of a PSP?

There are multiple global consensus guidelines, especially in Europe, on the indication for surgery [1, 4, 15, 106, 129].

A surgical management is therefore proposed:

During the second episode of PSP (ipsi- or contralateral) [39]. From the first episode in case of

- SHP [94],
- simultaneous bilateral PSP,
- PSP with signs of severity,
- persistent air leaks or persistent pneumothorax despite suction drainage [130]. The definition of "persistent air leaks/prolonged bubbling" varies in the literature from 2 to 14 days [1, 4], and often arbitrarily set at 5 days [106],
- risky occupation or leisure activity (pilot, isolated workplace) [131, 132] (see ESM),
- PSP occurring during pregnancy (surgery after birth) [133],
- patient’s request [4].

The benefit-on-risk ratio of the surgical procedure should be discussed with the patient. The reduced postoperative recurrence rate of 0–10% is weighted by the estimated surgical morbidity rate of 2.4–9% [134].

**The group recommends to perform pleurodesis after a second episode of PSP (ipsi- or contralateral) regardless of the management method used for the first episode. (Strong recommendation, low level of evidence)**

**The group suggests to perform pleurodesis from the first episode of PSP in the following cases:**

- **Haemopneumothorax,**
- **Simultaneous bilateral PSP,**
- **Presence of signs of severity,**
- **Persistent air leaks or persistent pneumothorax despite suction drainage**
- **Risky occupation or leisure activity (pilot, isolated workplace…),**
- **PSP occurring during pregnancy (surgery after birth). (Conditional recommendation, low level of evidence)**

**The group proposes to respond to the patient’s request for surgery after a first episode of PSP after informing him/her of the risks and benefits of pleurodesis. (Expert opinion)**

#### Which approach should be used to perform surgery?

The main approaches described in the surgical treatment of PSP include:

- Postero-lateral thoracotomy, with or without muscle sparing,
- Axillary thoracotomy,
- Multiportal thoracoscopy, with or without automated assistance,
- Uniportal intercostal or subxiphoid thoracoscopy.

Mechanical or chemical techniques of bulla resection and pleurodesis may be performed through these approaches [135]. Pleurectomy may be technically difficult to perform using some approaches, and studies lack precision on the extent of pleurectomy performed. The resection of large bullae is more challenging using some uniportal techniques [136].

Studies that have compared open and closed chest approaches are often outdated. In the only prospective study comparing thoracoscopy and posterolateral thoracotomy, thoracoscopy was associated with better outcomes in terms of pain, postoperative hospital length of stay, and decline in FEV1 [137]. According to a meta-analysis, the outcomes of axillary thoracotomy are similar to those of thoracoscopy except for the recurrence rate (3–4 times higher with thoracoscopy) [138].

Currently, there is no guideline in favor of thoracotomy [2, 15]. Thoracoscopy is the most used and recommended in Europe [15, 106, 129, 139, 140].

Regarding pleurodesis, thoracoscopy is therefore proposed as a first-line procedure in many European countries. However, recurrence rate which was significantly higher after thoracoscopy compared to thoracotomy [139–141].

Two small retrospective studies have assessed treatment of PSP recurrence after surgically-induced pleurodesis, with heterogeneous techniques [142, 143]. These studies support repeating the intervention to a medical treatment and the use of a thoracoscopic to a thoracotomic approach.

Pain management was assessed in a 2004 meta-analysis, including 6 RCTs in pneumothorax surgery [144]. All RCTs have reported a decrease in pain scores and a reduction in postoperative analgesic use in the thoracoscopy group (VATS) compared to the thoracotomy group. Two studies have also reported a shorter hospital length of stay [145, 146]. Since this meta-analysis, several studies have confirmed the beneficial impact of VATS on postoperative pain [147–149].

Regarding thoracoscopy, comparative studies are limited by the diversity of the technique used [150, 151]. Three closed-chest approaches have emerged: intercostal thoracoscopy through several trocars (multi-port), through a single trocar (single-port) [152] and thoracoscopy through an abdominal approach (subxyphoid approach) [153].

Studies comparing single-port *versus* multi-port procedures are scarce [150, 151, 154] and one was randomized [135]. The single-port approach is more challenging for complex cases [155], with a potentially higher conversion rate [136]. On the other hand, regarding pain management, 2 meta-analyzes have included cohort studies which were conducted specifically in surgically-treated pneumothorax [167, 172]. They concluded that single-port VATS was superior to the 3-port technique in terms of postoperative pain (pain scores, postoperative paraesthesia), and shorter hospital length of stay. Since the publication of these meta-analyzes, an RCT included patients who underwent single-port, 2-port surgery and 3-port surgery [151]: the postoperative pain scores were significantly lower 4, 24 and 72 h after surgery with a single-port approach.

The more recent subxiphoid approach allows the simultaneous treatment of bilateral bullae. Its risk of postoperative pain should be weighed with a higher risk of arrhythmia and abdominal complications such as eventration [153].

Finally, regarding thoracoscopy, a multi-port approach (better long-term outcomes, fewer conversions) or a single-port approach (reduced postoperative pain) can be chosen, depending on the complexity of the procedure.

**If pleurodesis is indicated in a patient with PSP, the group recommends to use a minimally invasive technique. (Strong recommendation, high level of evidence).**

#### What are the different techniques to induce pleurodesis in PSP?

The main therapeutic objectives are to treat a potential persistent air leak and to prevent recurrence. There are two conventional techniques: chemical and mechanical pleurodesis, which includes mechanical pleural abrasion and parietal pleurectomy.

##### Parenchymal resection

The primary objective of a pulmonary resection would be to perform an etiological treatment of the pneumothorax, especially if a perforated bulla is found intraoperatively. It is usually achieved by stapling or sectioning the lung parenchyma with an automatic forceps. In the absence of a detectable lesion, an atypical parenchymal resection at the apex may be performed [156], contributing to subsequent pleurodesis, and allowing pathological analysis of the underlying lung. The use of aerostatic material has been suggested in order to cover the stapling line, to reduce the risk of recurrence [157].

##### Mechanical pleurodesis

It is targeted on the parietal pleura, while preserving the pulmonary hilum, the mediastinal and diaphragmatic pleura. Pleural abrasion consists in irritating the parietal pleura by a fitted pad or vicryl plate, until obtaining a haemorrhagic ooze, inducing pleurodesis [158]. Pleurectomy is the removal of the parietal sheet of the pleura. Extensive pleurectomy, is very effective for pleurodesis, but leads to a higher number of postoperative complications (haemorrhage, respiratory complications and chronic pain) [159]. Pleural abrasion is the most commonly used technique, although there is little evidence that it is superior to pleurectomy in reducing the recurrence rate [160]. The RCT by Rena et al. showed low morbidity rates as compared to apical pleurectomy without difference in recurrence rates between procedures [161]. A meta-analysis confirmed these findings, with a higher rate of intraoperative bleeding and longer postoperative drainage in the pleurectomy group [162].

##### Chemical pleurodesis

It is performed by instilling an irritant into the pleural space to obtain an inflammatory response leading to adhesions between the two pleural sheets.

Various irritants might be used: talc, tetracycline antibiotics, iodopovidine, dextrose, silver nitrate and blood patch [163].

Talc is the most commonly used irritant in Europe. The incidence of pleural or lung cancer is similar after talc use and in the general population, especially since preparations are regulated by drug agencies and are free of asbestos and other impurities [164].

Talc insufflation allows the absence of recurrence in more than 90% of cases [165], and allows to repeat the performance of VATS [166, 167]. However, the use of talc limits the possibility of subsequent thoracic surgery.

##### Is mechanical pleurodesis superior to chemical pleurodesis?

When comparing different VATS techniques, including bullectomy alone, chemical pleurodesis alone, abrasion alone and pleurectomy alone to treat PSP [168], recurrence occurred in 1.4% of cases in the bullectomy combined with abrasion and in 0.4% of cases in the bullectomy combined with chemical pleurodesis. No recurrence was observed in the pleurectomy alone group. A cohort study of PSP treated with subtotal parietal pleurectomy via thoracoscopy or talc-induced pleurodesis [167] showed a recurrence rate of 9.15% in the pleurectomy group *vs.* 1.79% in the talc-induced pleurodesis group (P = 0.00018). However, the use of talc in a young subject rends a subsequent thoracic approach excessively complex.

##### Should multiple pleurodesis methods be combined to reduce the risk of recurrence?

A meta-analysis compared combined pleurodesis (mechanical and chemical) and mechanical pleurodesis alone in patients who underwent thoracoscopy [169]. In patients treated with a combined procedure, the risk of pneumothorax recurrence was reduced by 63% compared to those treated with a single pleurodesis technique. In contrast, they experienced increased chest pain, requiring higher doses of morphine analgesics.

Before performing pleurodesis, the diagnosis of SSP should be discussed and the possibility of subsequent thoracic surgery (lung transplantation in a chronic respiratory disease patient, or aortic surgery in a patient with initially undiagnosed Marfan disease) should be considered. The surgical procedure should be adapted accordingly.

**The group suggests to induce mechanical and/or chemical pleurodesis as a first-line procedure rather than to perform pleurectomy if there is a surgical indication for pleurodesis. (Conditional recommendation, moderate level of evidence).**

##### CTD procedures following pleurodesis

No consensus exists on postoperative CTD after pleurodesis. The aim is to achieve optimal pulmonary reexpansion allowing adhesion of the pleural layers without residual pneumothorax [170, 171].

##### Perioperative analgesia

Perioperative analgesia is one of the major aspects in the management of PSP: this was highlighted by the two participating expert patients of these guidelines.

*LRA and epidural thoracic anesthesia*. Postoperative LRA technique in thoracic surgery reduces the use of postoperative morphine derivatives regardless of the surgical approach, and limits the occurrence of postoperative chronic pain [172, 173].

**The group suggests to use a perioperative locoregional analgesia technique in pneumothorax surgery to reduce postoperative pain. (Conditional recommendation, moderate level of evidence).**

Peripheral LRA techniques (including paravertebral block) and epidural analgesia for thoracotomy have been compared. Four meta-analyzes supported an equivalent analgesic efficacy of the paravertebral block with a better tolerance than epidural analgesia [174–177]. These data are in line with 2 RCTs, either with a continuous paravertebral block in VATS [178] or serratus plane block after thoracotomy [179] compared to epidural analgesia, with a higher efficacy and fewer AEs. In PSP surgery with VATS, a prospective study failed to find any analgesic superiority of epidural as compared to systemic analgesia with morphine derivatives [180].

**The group suggests to prefer peripheral locoregional** **analgesia (paravertebral block, serratus plane block, intercostal block) over thoracic epidural analgesia. (Conditional** **recommendation, moderate level of evidence).**

*Non-steroidal anti-inflammatory drugs (NSAID) after pneumothorax surgery.* NSAID use after thoracic surgery is suggested as a component of multimodal analgesia to reduce pain and promote rehabilitation [181]. Their use reduced by half the number of patients treated with morphine derivatives at the time of discharge and one week after surgery [182].

Experimental studies in animal models suggested that NSAID administration would reduce the efficacy of surgical pleurodesis [183, 184], but has not been demonstrated to date in humans [182, 185, 186].

**The group suggests to use NSAID for a few days after pneumothorax surgery in case of insufficient locoregional analgesia and non-morphine systemic analgesia to reduce or prevent the use of morphine. The use of NSAID does not seem to decrease the efficacy of surgical pleurodesis. (Conditional recommendation, moderate level of evidence)**

### Post-PSP management

#### Smoking cessation

Smoking tobacco significantly increases the risk of a first SP, moreover when associated with cannabis [187], and is a risk factor for recurrence [188]. Conversely, smoking cessation is known to significantly reduce the risk of recurrence [14].

**The group recommends to offer tobacco-smoking (and any other smoked substances) cessation support to patients to minimize the risk of PSP recurrence. (Strong recommendation, high level of evidence).**

#### Follow-up after an episode of PSP and indication of chest CT-scan

The goals for the follow-up are: detecting an underlying disease, provide information on risk of recurrence, and promote smoking cessation

After a first episode, the risk of recurrence ranged between 0 and 67% [4] and more than half of the recurrences occurred during the first year [14]. The most frequent underlying chronic lung disease associated with a first episode of SP, are mainly COPD, emphysema and asthma [189]. These diseases may be suspected during a complete physical examination.

**The group proposes to schedule a consultation with a pulmonologist after each episode of PSP to detect any underlying lung disease. (Expert opinion)**

Secondary causes of pneumothorax can be detected by chest CT-scan such as cystic, interstitial, obstructive or collagen diseases, infections, catamenial pneumothorax, cancer, or other rare diseases [19, 190, 191]. A normal CXR can occult some abnormalities, such as emphysema [192], or multiple cystic lung diseases [193].

High-quality studies advocate against the use of CT-scan, as it does not impact the incidence of recurrences [194–196], or modify the management of a PSP [197, 198], but it increases costs and radiation exposure [199, 200].

The literature does not support the performance of a chest CT-scan after a first episode of unilateral PSP, in the absence of signs suggestive of a secondary cause [17], as the presence of an underlying respiratory disease might be predictive factors for recurrence [201–203]. However, bilateral PSP requires further investigation to determine the aetiology, and rule out an underlying respiratory disease [204].

**The group suggests not to systematically perform a chest CT-scan after a PSP, except in case of bilateral or recurrent PSP or in a context suggestive of an underlying lung disease. (Conditional recommendation, moderate level of evidence)**

### Precautions after a first episode of PSP

#### Air travel

The increase in altitude results in a decrease in atmospheric pressure and has direct impact on intrathoracic pressure, and the size of a pneumothorax [124]. Although the literature does not report any complications on airlifted patients with pneumothorax, either drained [123] or not [125, 205]. Previously published guidelines contraindicate flying when there is a radiological pneumothorax, or after a period of 7 days to 3 weeks [6, 131, 206].

**The group proposes to wait at least 2 weeks after PSP resolution before flying. (Expert opinion).**

**The group proposes to perform pleurodesis through the first episode of PSP in aircrew. (Expert opinion).**

#### Skydiving and freefall

Skydiving exposes to hypobaria and hypoxia due to high drop altitudes [207]. As with air travel, skydiving is absolutely contraindicated until pneumothorax resolution. No study has assessed the risk of SP during skydiving.

Only few cases of post-traumatic pneumothorax were reported during skydiving [208, 209].

The French Skydiving Federation contraindicates its practice in case of recurrent pneumothorax in the absence of pleurodesis history.

**The group proposes to perform pleurodesis after the first episode of PSP in sport skydivers and to perform a chest CT-scan and PFT before resuming their activity. (Expert opinion)**

#### Scuba diving with air tanks

The main complication of scuba diving is barotrauma, mainly resulting in pneumomediastinum, gas embolism and sometimes pneumothorax [210], which can be life-threatening.

Although no case of pneumothorax is reported in the analysis of diving accidents [211, 212], a history of PSP has long been considered as an absolute contraindication to scuba diving [213].

Pleurodesis and pleurectomy reduce, but does not eliminate, the risk of recurrence [214, 215].

**The group proposes to strongly contraindicate scuba diving in patients with a history of PSP, even if the patient has undergone pleurodesis, due to the risk of fatal barotrauma. (Expert opinion)**

#### Physical activities

Pneumothorax occurs at rest in more than 80% of cases [216], and sports does not seem to be a triggering factor [6].

**The group proposes not to limit sports resumption/practice after PSP resolution. (Expert opinion)**

#### Wind instruments

Only one clinical case of PSP in a non-professional trumpeter has been reported in the literature [217].

**The group proposes not to limit the practice of wind instruments after PSP resolution. (Expert opinion)**

### Unmet needs

Despite a growing, but sometimes outdated, literature, questions on the different steps of PSP management still exist.

#### Diagnostic imaging

The evolution of imaging techniques allows maintaining diagnostic performances of the CT-scan while decreasing radiation exposure. The place of ultra-low-dose chest CT-scan remains to be determined.

#### Monitoring and strategies for drainage device weaning

After CTD, the time to aspiration could be investigated with regard to anamnestic or clinical characteristics, with the aim of relieving symptoms or reducing the inpatient management duration. Similarly, the interest of clamping the chest tube during its weaning is still debated in medically-treated patients, and the interest of this procedure remains to be investigated.

Finally, new devices are emerging and deserve to be assessed [13].

#### Surgical management

Postoperative drainage duration is variable, and further studies are needed to determine the minimal drainage time to achieve satisfactory pleurodesis. The development of instrumentation (3-mm micro-incision) could result in a change in the current management techniques.

#### Analgesia for pleural procedures in PSP

The existing data on pain of PSP derives from studies of cardiothoracic surgery, as it has not been specifically assessed for PSP, while each population have specificities. The data on PSP are necessary: multimodal analgesia strategies, use of non-pharmacological techniques (music therapy, hypnosis, etc.), or physical techniques (cold). An update of historical data on pain after a PSP is essential with the use of small-bore devices. The participating expert patients of these guidelines, emphasized that the total absence of chest pain regardless of the therapeutic management method or location for PSP patients is necessary.

## Conclusion

These first French guidelines on PSP gathered all the professional groups involved in the management of PSP patients, and expert patients. They were approved by a Delphi consensus, and their robust methodology will hopefully lead to their widespread use. Beyond some assessments and management methods that are fairly consensual, we deliberately discussed some specific aspects with the aim of helping clinicians. In addition, in line with societal and healthcare system developments, our group strongly supported the outpatient management, but only if a prior well-defined organization is integrated in the care structure and in the city network.

Research on PSP is still ongoing and these guidelines are far from being set in stone. They could evolve in the upcoming years.

### Obituary

Dr Martinez died suddenly on the 24th of October 2022, at the age of 46-year-old. Our thoughts are with Mikaël Martinez and his family. Doctor Martinez was a brilliant mind, committed to emergency medicine and honourably served the public hospital during his career. We miss you, our friend.

**Table:** Summary of guidelines on PSP

Guidelines		Grade of recommendation	Level of evidence
2. Diagnostic strategy, assessment, follow-up method			
R 2.1	The group suggests to consider a PSP as large when there is a visible rim along the entire axillary line, ≥ 2 cm between the lung margin and the chest wall at the hilum level.	Conditional recommendation	Low
R 2.2.1	The group proposes that, although the chest CT-scan is superior to CXR for the positive diagnosis of PSP, assessing its size and ruling out a differential diagnosis, its cost, radiation exposure and accessibility do not support its use as a first-line examination.	Expert opinion	
R 2.2.1bis	The group proposes to perform frontal CXR acquired in inspiration, without expiratory films, in case of suspected PSP to diagnose it and assess its size.	Expert opinion	
R 2.2.2	The group recommends to perform a low-radiation chest CT-scan in case of persistent diagnostic doubt despite the investigations already performed.	Strong recommendation	Low
R 2.2.3	The group suggests not to solely base the diagnosis of PSP on chest ultrasound in the absence of signs of severity.	Conditional recommendation	Low
R 2.3	The group proposes not to solely base on chest ultrasound to assess the size of a PSP.	Expert opinion	
	No data in the literature allow concluding on the value of chest ultrasound to rule out the differential diagnoses of PSP.	NA	
R 2.4	The group suggests to perform chest ultrasound for the diagnosis of residual pneumothorax in patients drained for pneumothorax. In untrained teams or teams with limited access to ultrasound, CXR may be used as an alternative.	Conditional recommendation	Moderate
3. Therapeutic management			
Medical treatment of PSP			
R 3.1.1	The group recommends to consider a pneumothorax as tension when it results in respiratory distress or hemodynamic failure.	Strong recommendation	Low
R 3.1.2	In case of confirmed tension PSP, the group recommends: - to perform emergency chest decompression, - through an anterior (mid-clavicular line at the 2nd intercostal space) or axillary (mid-axillary line at the 4th intercostal space) approach, - using dedicated equipment (thoracentesis kit) or any other needle aspiration device available to the operator.	Strong recommendation	Low
R 3.2.1.1	The group recommends to remove air from the pleural space in patients with large PSP without signs of immediate severity.	Strong recommendation	Moderate
R 3.2.1.2	The group recommends to use either needle aspiration or chest tube drainage as first-line treatment in patients with large PSP without signs of immediate severity to remove air from the pleural space. Surgery should not be performed as first-line treatment except in specific situations (see chapter on surgery).	Strong recommendation	Moderate
R 3.2.1.4	The group recommends to prefer the outpatient management in patients with large PSP without signs of immediate severity.	Strong recommendation	Moderate
R 3.2.1.4bis	The group recommends an outpatient management based on needle aspiration or on the placement of a mini-chest tube and a one-way valve, if the following criteria are met: - the patient is stable after removal of the intrapleural air, - and a dedicated outpatient care system is previously organized, - and a consultation with chest ultrasound or CXR is scheduled at 24 − 72 h to follow the evolution.	Strong recommendation	Low
R 3.2.1.4ter	The group proposes to manage PSP on an outpatient basis only if all of the following conditions are met: - The patient has the procedure to be followed in case of problem 24 h a day, 7 days a week, with the appropriate phone numbers including calling the SAMU-Center 15 (provision of a standardized written document) - Ensuring that the patient has understood the guidelines in case of problems - The patient should not stay alone for the first 24 − 48 h after discharge - The patient should be able to access a medical facility within 1 h, regardless of the means of transportation, in the event of deterioration, The time of discharge does not matter if all of the above criteria are met (i.e. a deep night discharge is possible).	Expert opinion	
R 3.2.2	The group recommends to manage conservatively (monitoring) patients with small PSP and without signs of poor tolerance.	Strong recommendation	Low
R 3.2.2bis	The group recommends to implement an outpatient, conservative management for small PSP if the following criteria are met: - the patient is clinically and radiologically stable after 4 h, - and a dedicated outpatient care system is previously organized, - and a consultation with chest ultrasound or CXR is scheduled at 24 − 72 h to follow the evolution.	Strong recommendation	Low
R 3.4.1	In case of simultaneous bilateral PSP, regardless of its size, the group proposes to contact as soon as possible an expert center, i.e. a center with a thoracic surgery department, to discuss the treatment approach and consider a possible transfer to this center.	Expert opinion	
R 3.4.1bis	In case of simultaneous bilateral PSP with signs of severity or large PSP, the group proposes to perform emergency chest tube drainage.	Expert opinion	
R 3.4.2	In case of haemopneumothorax, regardless of its size, the group proposes to contact as soon as possible an expert center, i.e. a center with a thoracic surgery department, to discuss the treatment approach and consider a possible transfer to this center.	Expert opinion	
R 3.4.2bis	In case of haemopneumothorax with signs of severity or large haemopneumothorax, the group proposes to perform emergency chest tube drainage.	Expert opinion	
R 3.4.3	In case of PSP with confirmed pleural adhesion, regardless of its size, the group proposes to contact as soon as possible an expert center, i.e. a center with a thoracic surgery department, to discuss the treatment approach and consider a possible transfer to this center.	Expert opinion	
R 3.4.3bis	In case of PSP with confirmed pleural adhesion and signs of severity or large PSP, the group proposes to perform emergency chest tube drainage.	Expert opinion	
R 3.5.1	The group suggests to use a small-bore chest tube (≤ 14 Fr) for chest drain insertion of PSP.	Conditional recommendation	Low
R 3.5.2	The group suggests to obtain an ultrasound visualization before performing needle aspiration or chest tube drainage using the anterior or axillary approach, in order to reduce the risk of complications.	Conditional recommendation	Low
	The literature does not provide sufficient data to choose between the anterior and axillary approach.	NA	
R 3.5.3	The group recommends to initiate drainage with passive air removal (one-way valve or free flow) and to start suction at − 5 to − 20 cm H2O as a second step only if reexpansion is not achieved	Strong recommendation	Moderate
R 3.5.4	In patients under chest tube suction, in the absence of bubbling and with lung re-expansion, the group proposes to allow a free flow for 6 − 8 h before chest tube removal to avoid a new drainage procedure in case of early recurrence.	Expert opinion	
	The data in the literature do not allow concluding on the interest of performing a clamping trial before chest tube removal once the lung is re-expanded.	N/A	
R 3.6.1	The group recommends not to systematically administer oxygen therapy in patients treated for PSP.	Strong recommendation	Moderate
R 3.6.2	The group suggests not to prescribe strict bed rest in PSP patients.	Conditional recommendation	Low
R 3.6.2bis	The group proposes to limit intense or contact sports activities until complete resolution of the pneumothorax.	Expert opinion	
R 3.7	To reduce the risk of tension pneumothorax, the group proposes to arrange the transportation of patients with drained SP as follows: - In the absence of bubbling: with a chest tube attached to a one-way valve; - In the presence of bubbling: by continuing continuous suction with fitting of a stand-alone suction pump connected to the 3-compartment drainage system.	Expert opinion	
Surgical approach of PSP			
R 3.8.1	The group recommends to perform pleurodesis after a second episode of PSP (ipsi- or contralateral) regardless of the management method used for the first episode.	Strong recommendation	Low
R 3.8.1bis	The group suggests to perform pleurodesis through the first episode of PSP in the following cases: - Hemopneumothorax, - Simultaneous bilateral PSP, - Presence of signs of severity, - Persistent air leaks or persistent pneumothorax despite aspiration drainage, - Risky occupation or leisure activity (pilot, isolated workplace, etc.), - PSP occurring during pregnancy (surgery after birth),	Conditional recommendation	Low
R 3.8.1ter	The group proposes to respond to the patient’s request for surgery after a first episode of PSP after informing him/her of the risks and benefits of pleurodesis.	Expert opinion	
R 3.8.2	If pleurodesis is indicated in a patient with PSP, the group recommends to use a minimally invasive technique.	Strong recommendation	High
R 3.8.3	The group suggests to induce mechanical and/or chemical pleurodesis as a first-line procedure rather than to perform pleurectomy if there is a indication for pleurodesis surgery.	Conditional recommendation	Moderate
Analgesic treatment of PSP			
R 3.3.1	The group recommends to perform local anesthesia of the chest wall before removing air from the pleural space, either through needle aspiration or chest tube drainage.	Strong recommendation	Low
R 3.3.2	The group recommends to base pain management on multimodal analgesia in patients medically treated for PSP (needle aspiration, drainage, conservative management).	Strong recommendation	Low
R 3.3.3	The group recommends to use a multimodal analgesic approach including local cold treatment to reduce pain associated with large-bore chest tube removal (≥ 16 Fr) or in patients operated on for PSP.	Strong recommendation	Low
R 3.3.3bis	The group proposes to use analgesia during small-bore chest tube removal, but the current literature does not allow defining a preferred analgesia. Dedicated studies are needed.	Expert opinion	
R 3.8.4.1	The group suggests to use a perioperative locoregional analgesia technique in pneumothorax surgery to reduce postoperative pain.	Conditional recommendation	Moderate
R 3.8.4.1bis	The group suggests to prefer peripheral locoregional analgesia (paravertebral block, serratus plane block, intercostal block) over thoracic epidural analgesia.	Conditional recommendation	Moderate
R 3.8.4.2	The group suggests to use non-steroidal anti-inflammatory drugs for a few days after pneumothorax surgery in case of insufficient locoregional analgesia and non-morphine systemic analgesia to reduce or prevent the use of morphine. The use of non-steroidal anti-inflammatory drugs does not seem to decrease the efficacy of surgical pleurodesis.	Conditional recommendation	Moderate
4. Follow-up procedures			
R 4.1	The group recommends to offer smoking (and any other smoked substances) cessation support to patients to minimize the risk of PSP recurrence.	Strong recommendation	High
R 4.2	The group proposes to schedule a consultation with a pulmonologist after each episode of PSP to detect any underlying lung disease.	Expert opinion	
R 4.2bis	The group suggests not to systematically perform a chest CT-scan after a PSP, except in case of bilateral or recurrent PSP or in a context suggestive of an underlying disease (secondary spontaneous pneumothorax).	Conditional recommendation	Moderate
R 4.3.1	The group proposes to wait at least two weeks after PSP resolution before flying.	Expert opinion	
R 4.3.1bis	The group proposes to perform pleurodesis from the first episode of PSP in aircrew.	Expert opinion	
R 4.3.2	The group proposes to perform pleurodesis after the first episode of pneumothorax in sport skydivers and to perform a chest CT-scan and PFT before resuming their activity.	Expert opinion	
R 4.3.3	The group proposes to strongly contraindicate scuba diving in patients with a history of PSP, even if the patient has undergone pleurodesis, due to the risk of fatal barotrauma.	Expert opinion	
R 4.3.4	The group proposes not to limit sports resumption/practice after an episode of PSP.	Expert opinion	
R 4.3.5	The group proposes not to limit the practice of wind instruments after an episode of PSP.	Expert opinion	

### Supplementary Information

**Additional file 1: Appendix S1.** Table presenting the bibliographic search equations in PubMed. **Appendix S2.** Different methods for assessing pneumothorax size. **Figure S1.** Schematic representation of the method for assessing pneumothorax size: Rhea’s method. **Figure S2.** Schematic representation of the method for assessing pneumothorax size: Collins’ method. **Figure S3.** Schematic representation of the method for assessing pneumothorax size: Light’s method. **Appendix S3.** High-risk occupations requiring adjustments with regard to surgical indications

## Data Availability

Not applicable.

## Abbreviations

ACCP: American College of Chest Physicians
AE: Adverse Effect
BHD: Birt − Hogg − Dubé syndrome
BSP: Belgian Society of Pulmonology
BTS: British Thoracic Society
CEP: French College of Respiratory Teachers
COPD: Chronic Obstructive Pulmonary Disease
CT: Computed Tomography
CTD: Chest Tube Drainage
CXR: Chest XRay
ERS: European Respiratory Society
Fr: French
GRADE: Grading of Recommendation Assessment: Development and Evaluation
IPF: Idiopathic Pulmonary Fibrosis
LRA: Locoregional Analgesia
NA: Needle Aspiration
NSAID: Non-Steroidal Anti-Inflammatory Drug
OR: Odds Ratio
PFTs: Pulmonary Function Tests
PICO: Patients/Population, Intervention, Comparison, Outcomes
PSP: Primary Spontaneous Pneumothorax
SAE: Serious Adverse Effect
SEPAR: Spanish Society of Pulmonology and Thoracic Surgery
SFAR: French Society of Anesthesia & Intensive Care Medicine
SFCTCV: French Society of Thoracic and Cardiovascular Surgery
SFMU: French Society of Emergency Medicine
SNS: Simple Numerical Scale
SP: Spontaneous Pneumothorax
SPLF: French Speaking Society of Respiratory Diseases
SRLF: French Intensive Care Society
SSP: Secondary Spontaneous Pneumothorax
VAS: Visual Analog Scale
VATS: Video-Assisted Thoracoscopy

## References

[1] Aguinagalde B, Aranda JL, Busca P, Martinez I, Royo I, Zabaleta J (2018). SECT Clinical practice guideline on the management of patients with spontaneous pneumothorax. Cir Esp.

[2] Baumann MH, Strange C, Heffner JE, Light R, Kirby TJ, Klein J (2001). Management of spontaneous pneumothorax: an American college of chest physicians Delphi consensus statement. Chest.

[3] De Leyn P, Lismonde M, Ninane V, Noppen M, Slabbynck H, Van Meerhaeghe A (2005). Guidelines belgian society of pneumology guidelines on the management of spontaneous pneumothorax. Acta Chir Belg.

[4] MacDuff A, Arnold A, Harvey J, Group BTSPDG (2010). Management of spontaneous pneumothorax: British thoracic society pleural disease guideline 2010. Thorax.

[5] Collins CD, Lopez A, Mathie A, Wood V, Jackson JE, Roddie ME (1995). Quantification of pneumothorax size on chest radiographs using interpleural distances: regression analysis based on volume measurements from helical CT. AJR Am J Roentgenol.

[6] CEP. Conduite à tenir devant un pneumothorax. Référentiel du Collège des Enseignants de Pneumologie—7ème édition. 2021. http://cep.splf.fr/edition2021-du-referentiel-du-college-des-enseignants-de-pneumologie-cep-pour-lapreparation-des-ecn-7eme-edition/.

[7] Marquette CH, Marx A, Leroy S, Vaniet F, Ramon P, Caussade S (2006). Simplified stepwise management of primary spontaneous pneumothorax: a pilot study. Eur Respir J.

[8] Massongo M, Leroy S, Scherpereel A, Vaniet F, Dhalluin X, Chahine B (2014). Outpatient management of primary spontaneous pneumothorax: a prospective study. Eur Respir J.

[9] Sale A, Sohier L, Campion M, Le Ho R, Bazin Y, Gangloff C (2020). Exclusive ambulatory management of spontaneous pneumothorax with pigtail catheters, a prospective multicentric study. Respir Med.

[10] Voisin F, Sohier L, Rochas Y, Kerjouan M, Ricordel C, Belleguic C (2014). Ambulatory management of large spontaneous pneumothorax with pigtail catheters. Ann Emerg Med.

[11] Jouneau S, Vuillard C, Sale A, Bazin Y, Sohier L, Kerjouan M (2020). Outpatient management of primary spontaneous pneumothorax. Respir Med.

[12] Brown SGA, Ball EL, Perrin K, Asha SE, Braithwaite I, Egerton-Warburton D (2020). Conservative versus interventional treatment for spontaneous pneumothorax. N Engl J Med.

[13] Hallifax RJ, McKeown E, Sivakumar P, Fairbairn I, Peter C, Leitch A (2020). Ambulatory management of primary spontaneous pneumothorax: an open-label, randomised controlled trial. Lancet.

[14] Walker SP, Bibby AC, Halford P, Stadon L, White P, Maskell NA (2018). Recurrence rates in primary spontaneous pneumothorax: a systematic review and meta-analysis. Eur Respir J.

[15] Tschopp JM, Bintcliffe O, Astoul P, Canalis E, Driesen P, Janssen J (2015). ERS task force statement: diagnosis and treatment of primary spontaneous pneumothorax. Eur Respir J.

[16] Kelly AM, Druda D (2008). Comparison of size classification of primary spontaneous pneumothorax by three international guidelines: a case for international consensus?. Respir Med.

[17] Ball CG, Kirkpatrick AW, Laupland KB, Fox DL, Litvinchuk S, Dyer DM (2005). Factors related to the failure of radiographic recognition of occult posttraumatic pneumothoraces. Am J Surg.

[18] Noh TJ, Lee CH, Kang YA, Kwon SY, Yoon HI, Kim TJ (2009). Chest computed tomography (CT) immediately after CT-guided transthoracic needle aspiration biopsy as a predictor of overt pneumothorax. Korean J Intern Med.

[19] Hilliard NJ, Marciniak SJ, Babar JL, Balan A (2013). Evaluation of secondary spontaneous pneumothorax with multidetector CT. Clin Radiol.

[20] Frankel HL, Kirkpatrick AW, Elbarbary M, Blaivas M, Desai H, Evans D (2015). Guidelines for the appropriate use of bedside general and cardiac ultrasonography in the evaluation of critically Ill patients-Part I: general ultrasonography. Crit Care Med.

[21] Ding W, Shen Y, Yang J, He X, Zhang M (2011). Diagnosis of pneumothorax by radiography and ultrasonography: a meta-analysis. Chest.

[22] Abdalla W, Elgendy M, Abdelaziz AA, Ammar MA (2016). Lung ultrasound versus chest radiography for the diagnosis of pneumothorax in critically ill patients: a prospective, single-blind study. Saudi J Anaesth.

[23] Blaivas M, Lyon M, Duggal S (2005). A prospective comparison of supine chest radiography and bedside ultrasound for the diagnosis of traumatic pneumothorax. Acad Emerg Med.

[24] Kaya S, Cevik AA, Acar N, Doner E, Sivrikoz C, Ozkan R (2015). A study on the evaluation of pneumothorax by imaging methods in patients presenting to the emergency department for blunt thoracic trauma. Ulus Travma Acil Cerrahi Derg.

[25] Sartori S, Tombesi P, Trevisani L, Nielsen I, Tassinari D, Abbasciano V (2007). Accuracy of transthoracic sonography in detection of pneumothorax after sonographically guided lung biopsy: prospective comparison with chest radiography. AJR Am J Roentgenol.

[26] Soult MC, Weireter LJ, Britt RC, Collins JN, Novosel TJ, Reed SF (2015). Can routine trauma bay chest x-ray be bypassed with an extended focused assessment with sonography for trauma examination?. Am Surg.

[27] Wilkerson RG, Stone MB (2010). Sensitivity of bedside ultrasound and supine anteroposterior chest radiographs for the identification of pneumothorax after blunt trauma. Acad Emerg Med.

[28] Jalli R, Sefidbakht S, Jafari SH (2013). Value of ultrasound in diagnosis of pneumothorax: a prospective study. Emerg Radiol.

[29] Tasci O, Hatipoglu ON, Cagli B, Ermis V (2016). Sonography of the chest using linear-array versus sector transducers: correlation with auscultation, chest radiography, and computed tomography. J Clin Ultrasound.

[30] Volpicelli G, Elbarbary M, Blaivas M, Lichtenstein DA, Mathis G, Kirkpatrick AW (2012). International evidence-based recommendations for point-of-care lung ultrasound. Intensive Care Med.

[31] Oveland NP, Soreide E, Lossius HM, Johannessen F, Wemmelund KB, Aagaard R (2013). The intrapleural volume threshold for ultrasound detection of pneumothoraces: an experimental study on porcine models. Scand J Trauma Resusc Emerg Med.

[32] Soldati G, Testa A, Sher S, Pignataro G, La Sala M, Silveri NG (2008). Occult traumatic pneumothorax: diagnostic accuracy of lung ultrasonography in the emergency department. Chest.

[33] Martino K, Merrit S, Boyakye K, Sernas T, Koller C, Hauser CJ (1999). Prospective randomized trial of thoracostomy removal algorithms. J Trauma.

[34] Karagoz A, Unluer EE, Akcay O, Kadioglu E (2018). Effectiveness of bedside lung ultrasound for clinical follow-up of primary spontaneous pneumothorax patients treated with tube thoracostomy. Ultrasound Q.

[35] Galbois A, Ait-Oufella H, Baudel JL, Kofman T, Bottero J, Viennot S (2010). Pleural ultrasound compared with chest radiographic detection of pneumothorax resolution after drainage. Chest.

[36] Barton ED (1999). Tension pneumothorax. Curr Opin Pulm Med.

[37] Roberts DJ, Leigh-Smith S, Faris PD, Blackmore C, Ball CG, Robertson HL (2015). Clinical presentation of patients with tension pneumothorax: a systematic review. Ann Surg.

[38] Kepka S, Dalphin JC, Pretalli JB, Parmentier AL, Lauque D, Trebes G (2019). How spontaneous pneumothorax is managed in emergency departments: a French multicentre descriptive study. BMC Emerg Med.

[39] Brown SG, Ball EL, Macdonald SP, Wright C, Mc DTD (2014). Spontaneous pneumothorax; a multicentre retrospective analysis of emergency treatment, complications and outcomes. Intern Med J.

[40] Kelly AM, Kerr D, Clooney M (2008). Outcomes of emergency department patients treated for primary spontaneous pneumothorax. Chest.

[41] Andrivet P, Djedaini K, Teboul JL, Brochard L, Dreyfuss D (1995). Spontaneous pneumothorax. Comparison of thoracic drainage vs immediate or delayed needle aspiration. Chest.

[42] Camuset J, Laganier J, Brugiere O, Dauriat G, Jebrak G, Thabut G (2006). Needle aspiration as first-line management of primary spontaneous pneumothorax. Presse Med.

[43] Vuillard C, Dib F, Achamlal J, Gaudry S, Roux D, Chemouny M (2019). Longer symptom onset to aspiration time predicts success of needle aspiration in primary spontaneous pneumothorax. Thorax.

[44] Ayed AK, Chandrasekaran C, Sukumar M (2006). Aspiration versus tube drainage in primary spontaneous pneumothorax: a randomised study. Eur Respir J.

[45] Chen JS, Tsai KT, Hsu HH, Yuan A, Chen WJ, Lee YC (2008). Intrapleural minocycline following simple aspiration for initial treatment of primary spontaneous pneumothorax. Respir Med.

[46] Harvey J, Prescott RJ (1994). Simple aspiration versus intercostal tube drainage for spontaneous pneumothorax in patients with normal lungs. British thoracic society research committee. BMJ.

[47] Nishiuma T, Ohnishi H, Katsurada N, Yamamoto S, Yoshimura S, Kinami S (2012). Evaluation of simple aspiration therapy in the initial treatment for primary spontaneous pneumothorax. Intern Med.

[48] Noppen M, Alexander P, Driesen P, Slabbynck H, Verstraeten A (2002). Manual aspiration versus chest tube drainage in first episodes of primary spontaneous pneumothorax: a multicenter, prospective, randomized pilot study. Am J Respir Crit Care Med.

[49] Parlak M, Uil SM, van den Berg JW (2012). A prospective, randomised trial of pneumothorax therapy: manual aspiration versus conventional chest tube drainage. Respir Med.

[50] Thelle A, Gjerdevik M, SueChu M, Hagen OM, Bakke P (2017). Randomised comparison of needle aspiration and chest tube drainage in spontaneous pneumothorax. Eur Respir J.

[51] Korczynski P, Gorska K, Nasilowski J, Chazan R, Krenke R (2015). Comparison of small bore catheter aspiration and chest tube drainage in the management of spontaneous pneumothorax. Adv Exp Med Biol.

[52] Chan SS, Lam PK (2005). Simple aspiration as initial treatment for primary spontaneous pneumothorax: results of 91 consecutive cases. J Emerg Med.

[53] Ho KK, Ong ME, Koh MS, Wong E, Raghuram J (2011). A randomized controlled trial comparing minichest tube and needle aspiration in outpatient management of primary spontaneous pneumothorax. Am J Emerg Med.

[54] Ganaie MB, Maqsood U, Lea S, Bankart MJ, Bikmalla S, Afridi MA (2019). How should complete lung collapse secondary to primary spontaneous pneumothorax be managed?. Clin Med (Lond).

[55] Ramouz A, Lashkari MH, Fakour S, Rasihashemi SZ (2018). Randomized controlled trial on the comparison of chest tube drainage and needle aspiration in the treatment of primary spontaneous pneumothorax. Pak J Med Sci.

[56] Chan JW, Ko FW, Ng CK, Yeung AW, Yee WK, So LK (2009). Management of patients admitted with pneumothorax: a multi-centre study of the practice and outcomes in Hong Kong. Hong Kong Med J.

[57] Kim IH, Kang DK, Min HK, Hwang YH (2019). A prospective randomized trial comparing manual needle aspiration to closed thoracostomy as an initial treatment for the first episode of primary spontaneous pneumothorax. Korean J Thorac Cardiovasc Surg.

[58] Contou D, Razazi K, Katsahian S, Maitre B, Mekontso-Dessap A, Brun-Buisson C (2012). Small-bore catheter versus chest tube drainage for pneumothorax. Am J Emerg Med.

[59] Carson-Chahhoud KV, Wakai A, van Agteren JE, Smith BJ, McCabe G, Brinn MP (2017). Simple aspiration versus intercostal tube drainage for primary spontaneous pneumothorax in adults. Cochrane Database Syst Rev.

[60] Olesen WH, Katballe N, Sindby JE, Titlestad IL, Andersen PE, Lindahl-Jacobsen R (2018). Surgical treatment versus conventional chest tube drainage in primary spontaneous pneumothorax: a randomized controlled trial. Eur J Cardiothorac Surg.

[61] Al-Mourgi M, Alshehri F (2015). Video-assisted thoracoscopic surgery for the treatment of first-time spontaneous pneumothorax versus conservative treatment. Int J Health Sci (Qassim).

[62] Hofmann HS, Suttner T, Neu R, Potzger T, Szoke T, Grosser C (2018). Burden between undersupply and overtreatment in the care of primary spontaneous pneumothorax. Thorac Cardiovasc Surg.

[63] Divisi D, Di Leonardo G, Crisci R (2015). Video-assisted thoracic surgery versus pleural drainage in the management of the first episode of primary spontaneous pneumothorax. Am J Surg.

[64] Torresini G, Vaccarili M, Divisi D, Crisci R (2001). Is video-assisted thoracic surgery justified at first spontaneous pneumothorax?. Eur J Cardiothorac Surg.

[65] Chen JS, Hsu HH, Tsai KT, Yuan A, Chen WJ, Lee YC (2008). Salvage for unsuccessful aspiration of primary pneumothorax: thoracoscopic surgery or chest tube drainage?. Ann Thorac Surg.

[66] Hassani B, Foote J, Borgundvaag B (2009). Outpatient management of primary spontaneous pneumothorax in the emergency department of a community hospital using a small-bore catheter and a Heimlich valve. Acad Emerg Med.

[67] Strazar AR, Leynes PG, Lalonde DH (2013). Minimizing the pain of local anesthesia injection. Plast Reconstr Surg.

[68] Engdahl O, Boe J, Sandstedt S (1993). Interpleural bupivacaine for analgesia during chest drainage treatment for pneumothorax. A randomized double-blind study. Acta Anaesthesiol Scand.

[69] Kol E, Erdogan A, Karsli B, Erbil N (2013). Evaluation of the outcomes of ice application for the control of pain associated with chest tube irritation. Pain Manag Nurs.

[70] Paiement B, Boulanger M, Jones CW, Roy M (1979). Intubation and other experiences in cardiac surgery: the consumer’s views. Can Anaesth Soc J.

[71] Demir Y, Khorshid L (2010). The effect of cold application in combination with standard analgesic administration on pain and anxiety during chest tube removal: a single-blinded, randomized, double-controlled study. Pain Manag Nurs.

[72] Payami MB, Daryei N, Mousavinasab N, Nourizade E (2014). Effect of cold application in combination with Indomethacin suppository on chest tube removal pain in patients undergoing open heart surgery. Iran J Nurs Midwifery Res.

[73] Gorji HM, Nesami BM, Ayyasi M, Ghafari R, Yazdani J (2014). Comparison of ice packs application and relaxation therapy in pain reduction during chest tube removal following cardiac surgery. N Am J Med Sci.

[74] Mohammadi N, Pooria A, Yarahmadi S, Tarrahi MJ, Najafizadeh H, Abbasi P (2018). Effects of cold application on chest tube removal pain in heart surgery patients. Tanaffos.

[75] Sauls J (2002). The use of ice for pain associated with chest tube removal. Pain Manag Nurs.

[76] Hsieh LY, Chen YR, Lu MC (2017). Efficacy of cold application on pain during chest tube removal: a randomized controlled trial: a CONSORT-compliant article. Medicine (Baltimore).

[77] Aktas YY, Karabulut N (2019). The use of cold therapy, music therapy and lidocaine spray for reducing pain and anxiety following chest tube removal. Complement Ther Clin Pract.

[78] Ertug N, Ulker S (2012). The effect of cold application on pain due to chest tube removal. J Clin Nurs.

[79] Blacha MMJ, Smesseim I, van der Lee I, van den Aardweg JG, Schultz MJ, Kik MLJ (2021). The legend of the buffalo chest. Chest.

[80] Lee SC, Cheng YL, Huang CW, Tzao C, Hsu HH, Chang H (2008). Simultaneous bilateral primary spontaneous pneumothorax. Respirology.

[81] Sayar A, Turna A, Metin M, Kucukyagci N, Solak O, Gurses A (2004). Simultaneous bilateral spontaneous pneumothorax report of 12 cases and review of the literature. Acta Chir Belg.

[82] Sunam G, Gok M, Ceran S, Solak H (2004). Bilateral pneumothorax: a retrospective analysis of 40 patients. Surg Today.

[83] Ayed AK (2002). Bilateral video-assisted thoracoscopic surgery for bilateral spontaneous pneumothorax. Chest.

[84] Lang-Lazdunski L, de Kerangal X, Pons F, Jancovici R (2000). Primary spontaneous pneumothorax: one-stage treatment by bilateral videothoracoscopy. Ann Thorac Surg.

[85] Wu YC, Chu Y, Liu YH, Yeh CH, Chen TP, Liu HP (2003). Thoracoscopic ipsilateral approach to contralateral bullous lesion in patients with bilateral spontaneous pneumothorax. Ann Thorac Surg.

[86] Yim AP (1996). Simultaneous vs staged bilateral video-assisted thoracoscopic surgery. Surg Endosc.

[87] Homma T, Sugiyama S, Kotoh K, Doki Y, Tsuda M, Misaki T (2009). Early surgery for treatment of spontaneous hemopneumothorax. Scand J Surg.

[88] Ng CS, Wong RH, Wan IY, Lau RW, Hsin MK, Yeung EC (2011). Spontaneous haemopneumothorax: current management. Postgrad Med J.

[89] Tatebe S, Kanazawa H, Yamazaki Y, Aoki E, Sakurai Y (1996). Spontaneous hemopneumothorax. Ann Thorac Surg.

[90] Kim ES, Kang JY, Pyo CH, Jeon EY, Lee WB (2008). 12-year experience of spontaneous hemopneumothorax. Ann Thorac Cardiovasc Surg.

[91] Boersma WG, Stigt JA, Smit HJ (2010). Treatment of haemothorax. Respir Med.

[92] de Perrot M, Deleaval J, Robert J, Spiliopoulos A (2000). Spontaneous hemopneumothorax−results of conservative treatment. Swiss Surg.

[93] Inafuku K, Maehara T, Yamamoto T, Masuda M (2015). Assessment of spontaneous hemopneumothorax: indications for surgery. Asian Cardiovasc Thorac Ann.

[94] Luh SP, Tsao TC (2007). Video-assisted thoracic surgery for spontaneous haemopneumothorax. Respirology.

[95] Chang YT, Dai ZK, Kao EL, Chuang HY, Cheng YJ, Chou SH (2007). Early videoassisted thoracic surgery for primary spontaneous hemopneumothorax. World J Surg.

[96] Singh S, Sharma ML, Lone RA, Wani MA, Hussain Z, Mir I (2009). Idiopathic massive spontaneous hemothorax: adhesion disruption. World J Surg.

[97] Benton IJ, Benfield GF (2009). Comparison of a large and small-calibre tube drain for managing spontaneous pneumothoraces. Respir Med.

[98] Iepsen UW, Ringbaek T (2013). Small-bore chest tubes seem to perform better than larger tubes in treatment of spontaneous pneumothorax. Dan Med J.

[99] Lai SM, Tee AK (2012). Outpatient treatment of primary spontaneous pneumothorax using a small-bore chest drain with a Heimlich valve: the experience of a Singapore emergency department. Eur J Emerg Med.

[100] Makris D, Marquette CH (2009). Drainage de la plèvre: les techniques et leurs pièges. Réanimation.

[101] Salamonsen M, Dobeli K, McGrath D, Readdy C, Ware R, Steinke K (2013). Physician-performed ultrasound can accurately screen for a vulnerable intercostal artery prior to chest drainage procedures. Respirology.

[102] Gray EJ, Cranford JA, Betcher JA, Huang RD, Kessler RA, Theyyunni N (2020). Sonogram of safety: ultrasound outperforms the fifth intercostal space landmark for tube thoracostomy site selection. J Clin Ultrasound.

[103] Brims FJ, Maskell NA (2013). Ambulatory treatment in the management of pneumothorax: a systematic review of the literature. Thorax.

[104] Beckett A, Savage E, Pannell D, Acharya S, Kirkpatrick A, Tien HC (2011). Needle decompression for tension pneumothorax in tactical combat casualty care: do catheters placed in the midaxillary line kink more often than those in the midclavicular line?. J Trauma.

[105] Shiroshita A, Matsui H, Yoshida K, Shiraishi A, Tanaka Y, Nakashima K (2020). Safety of the anterior approach versus the lateral approach for chest tube insertion by residents treating spontaneous pneumothorax: a propensity score weighted analysis. Gen Thorac Cardiovasc Surg.

[106] Schnell J, Beer M, Eggeling S, Gesierich W, Gottlieb J, Herth FJF (2019). Management of spontaneous pneumothorax and post-interventional pneumothorax: German S3 guideline. Respiration.

[107] So SY, Yu DY (1982). Catheter drainage of spontaneous pneumothorax: suction or no suction, early or late removal?. Thorax.

[108] Reed MF, Lyons JM, Luchette FA, Neu JA, Howington JA (2007). Preliminary report of a prospective, randomized trial of underwater seal for spontaneous and iatrogenic pneumothorax. J Am Coll Surg.

[109] Kepka S, Lemaitre L, Marx T, Bilbault P, Desmettre T (2019). A common gesture with a rare but potentially severe complication: re-expansion pulmonary edema following chest tube drainage. Respir Med Case Rep.

[110] Rozenman J, Yellin A, Simansky DA, Shiner RJ (1996). Re-expansion pulmonary oedema following spontaneous pneumothorax. Respir Med.

[111] Yoon JS, Suh JH, Choi SY, Kwon JB, Lee BY, Lee SH (2013). Risk factors for the development of reexpansion pulmonary edema in patients with spontaneous pneumothorax. J Cardiothorac Surg.

[112] Verhagen M, van Buijtenen JM, Geeraedts LM (2015). Reexpansion pulmonary edema after chest drainage for pneumothorax: a case report and literature overview. Respir Med Case Rep.

[113] Miller WC, Toon R, Palat H, Lacroix J (1973). Experimental pulmonary edema following re-expansion of pneumothorax. Am Rev Respir Dis.

[114] Becker JC, Zakaluzny SA, Keller BA, Galante JM, Utter GH (2020). Clamping trials prior to thoracostomy tube removal and the need for subsequent invasive pleural drainage. Am J Surg.

[115] Funk GA, Petrey LB, Foreman ML (2009). Clamping thoracostomy tubes: a heretical notion?. Proc (Bayl Univ Med Cent).

[116] Rasheed MA, Majeed FA, Ali Shah SZ, Naz A (2016). Role of clamping tube thoracostomy prior to removal in non-cardiac thoracic trauma. J Ayub Med Coll Abbottabad.

[117] Northfield TC (1971). Oxygen therapy for spontaneous pneumothorax. Br Med J.

[118] Park CB, Moon MH, Jeon HW, Cho DG, Song SW, Won YD (2017). Does oxygen therapy increase the resolution rate of primary spontaneous pneumothorax?. J Thorac Dis.

[119] Shaireen H, Rabi Y, Metcalfe A, Kamaluddeen M, Amin H, Akierman A (2014). Impact of oxygen concentration on time to resolution of spontaneous pneumothorax in term infants: a population based cohort study. BMC Pediatr.

[120] Clark SD, Saker F, Schneeberger MT, Park E, Sutton DW, Littner Y (2014). Administration of 100% oxygen does not hasten resolution of symptomatic spontaneous pneumothorax in neonates. J Perinatol.

[121] Brown SG, Ball EL, Perrin K, Read CA, Asha SE, Beasley R (2016). Study protocol for a randomised controlled trial of invasive versus conservative management of primary spontaneous pneumothorax. BMJ Open.

[122] Kim IS, Kim JJ, Han JW, Jeong SC, Kim YH (2020). Conservative treatment for recurrent secondary spontaneous pneumothorax in patients with a long recurrence-free interval. J Thorac Dis.

[123] Bunch A, Duchateau FX, Verner L, Truwit J, O’Connor R, Brady W (2013). Commercial air travel after pneumothorax: a review of the literature. Air Med J.

[124] Knotts D, Arthur AO, Holder P, Herrington T, Thomas SH (2013). Pneumothorax volume expansion in helicopter emergency medical services transport. Air Med J.

[125] Tam A, Singh P, Ensor JE, Carter K, Kim ES, Hicks ME (2011). Air travel after biopsy-related pneumothorax: is it safe to fly?. J Vasc Interv Radiol.

[126] Gogakos A, Barbetakis N, Lazaridis G, Papaiwannou A, Karavergou A, Lampaki S (2015). Heimlich valve and pneumothorax. Ann Transl Med.

[127] Heimlich HJ (1983). Heimlich valve for chest drainage. Med Instrum.

[128] Braude D, Tutera D, Tawil I, Pirkl G (2014). Air transport of patients with pneumothorax: is tube thoracostomy required before flight?. Air Med J.

[129] Mendogni P, Vannucci J, Ghisalberti M, Anile M, Aramini B, Congedo MT (2020). Epidemiology and management of primary spontaneous pneumothorax: a systematic review. Interact Cardiovasc Thorac Surg.

[130] Chee CB, Abisheganaden J, Yeo JK, Lee P, Huan PY, Poh SC (1998). Persistent airleak in spontaneous pneumothorax—clinical course and outcome. Respir Med.

[131] Hu X, Cowl CT, Baqir M, Ryu JH (2014). Air travel and pneumothorax. Chest.

[132] British Thoracic Society Fitness to Dive Group SotBTSSoCC (2003). British thoracic society guidelines on respiratory aspects of fitness for diving. Thorax.

[133] Lal A, Anderson G, Cowen M, Lindow S, Arnold AG (2007). Pneumothorax and pregnancy. Chest.

[134] Sihoe AD, Au SS, Cheung ML, Chow IK, Chu KM, Law CY (2004). Incidence of chest wall paresthesia after video-assisted thoracic surgery for primary spontaneous pneumothorax. Eur J Cardiothorac Surg.

[135] Kutluk AC, Kocaturk CI, Akin H, Erdogan S, Bilen S, Karapinar K (2018). Which is the best minimal invasive approach for the treatment of spontaneous pneumothorax? Uniport, two, or three ports: a prospective randomized trial. Thorac Cardiovasc Surg.

[136] Masmoudi H, Etienne H, Sylvestre R, Evrard D, Ouede R, Le Roux M (2017). Three hundred fifty-one patients with pneumothorax undergoing uniportal (single port) video-assisted thoracic surgery. Ann Thorac Surg.

[137] Waller DA, Forty J, Morritt GN (1994). Video-assisted thoracoscopic surgery versus thoracotomy for spontaneous pneumothorax. Ann Thorac Surg.

[138] Barker A, Maratos EC, Edmonds L, Lim E (2007). Recurrence rates of video-assisted thoracoscopic versus open surgery in the prevention of recurrent pneumothoraces: a systematic review of randomised and non-randomised trials. Lancet.

[139] Delpy JP, Pages PB, Mordant P, Falcoz PE, Thomas P, Le Pimpec-Barthes F (2016). Surgical management of spontaneous pneumothorax: are there any prognostic factors influencing postoperative complications?. Eur J Cardiothorac Surg.

[140] Asghar Nawaz M, Apparau D, Zacharias J, Shackcloth M (2019). Approach to pneumothorax surgery: a national survey of current UK practice. Asian Cardiovasc Thorac Ann.

[141] Fujiwara T, Tanaka K, Toyoda T, Inage T, Sakairi Y, Ishibashi F (2020). Risk factors of postoperative recurrence of primary spontaneous pneumothorax. J Thorac Dis.

[142] Cardillo G, Facciolo F, Regal M, Carbone L, Corzani F, Ricci A (2001). Recurrences following videothoracoscopic treatment of primary spontaneous pneumothorax: the role of redo-videothoracoscopy. Eur J Cardiothorac Surg.

[143] Chen JS, Hsu HH, Kuo SW, Huang PM, Lee JM, Lee YC (2009). Management of recurrent primary spontaneous pneumothorax after thoracoscopic surgery: should observation, drainage, redo thoracoscopy, or thoracotomy be used?. Surg Endosc.

[144] Sedrakyan A, van der Meulen J, Lewsey J, Treasure T (2004). Video assisted thoracic surgery for treatment of pneumothorax and lung resections: systematic review of randomised clinical trials. BMJ.

[145] Abdala OA, Levy RR, Bibiloni RH, Viso HD, De Souza M, Satler VH (2001). Advantages of video assisted thoracic surgery in the treatment of spontaneous pneumothorax. Medicina (B Aires).

[146] Gebhard FT, Becker HP, Gerngross H, Bruckner UB (1996). Reduced inflammatory response in minimal invasive surgery of pneumothorax. Arch Surg.

[147] Olavarrieta JR, Coronel P (2009). Expectations and patient satisfaction related to the use of thoracotomy and video-assisted thoracoscopic surgery for treating recurrence of spontaneous primary pneumothorax. J Bras Pneumol.

[148] Qureshi R, Nugent A, Hayat J, Qureshi M, Norton R (2008). Should surgical pleurectomy for spontaneous pneumothorax be always thoracoscopic?. Interact Cardiovasc Thorac Surg.

[149] Foroulis CN, Anastasiadis K, Charokopos N, Antonitsis P, Halvatzoulis HV, Karapanagiotidis GT (2012). A modified two-port thoracoscopic technique versus axillary minithoracotomy for the treatment of recurrent spontaneous pneumothorax: a prospective randomized study. Surg Endosc.

[150] Qin SL, Huang JB, Yang YL, Xian L (2015). Uniportal versus three-port video-assisted thoracoscopic surgery for spontaneous pneumothorax: a meta-analysis. J Thorac Dis.

[151] Xu W, Wang Y, Song J, Mo L, Jiang T (2017). One-port video-assisted thoracic surgery versus three-port video-assisted thoracic surgery for primary spontaneous pneumothorax: a meta-analysis. Surg Endosc.

[152] Perna V, Carvajal AF, Torrecilla JA, Gigirey O (2017). Scientific rigour must come first. Eur J Cardiothorac Surg.

[153] Chen L, Liu F, Wang B, Wang K (2019). Subxiphoid vs transthoracic approach thoracoscopic surgery for spontaneous pneumothorax: a propensity score-matched analysis. BMC Surg.

[154] Salati M, Brunelli A, Xiume F, Refai M, Sciarra V, Soccetti A (2008). Uniportal video-assisted thoracic surgery for primary spontaneous pneumothorax: clinical and economic analysis in comparison to the traditional approach. Interact Cardiovasc Thorac Surg.

[155] Gonfiotti A, Jaus MO, Barale D, Viggiano D, Battisti N, Macchiarini P (2015). Uniportal videothoracoscopic surgery: our indications and limits. Innovations (Phila).

[156] Jeon HW, Kim YD, Sim SB (2020). Should we consider the resected lung volume in primary spontaneous pneumothorax?. World J Surg.

[157] Lee S, Kim HR, Cho S, Huh DM, Lee EB, Ryu KM (2014). Staple line coverage after bullectomy for primary spontaneous pneumothorax: a randomized trial. Ann Thorac Surg.

[158] Gossot D, Galetta D, Stern JB, Debrosse D, Caliandro R, Girard P (2004). Results of thoracoscopic pleural abrasion for primary spontaneous pneumothorax. Surg Endosc.

[159] Ocakcioglu I, Kupeli M (2019). Surgical treatment of spontaneous pneumothorax: pleural abrasion or pleurectomy?. Surg Laparosc Endosc Percutan Tech.

[160] Park JS, Han WS, Kim HK, Choi YS (2012). Pleural abrasion for mechanical pleurodesis in surgery for primary spontaneous pneumothorax: is it effective?. Surg Laparosc Endosc Percutan Tech.

[161] Rena O, Massera F, Papalia E, Della Pona C, Robustellini M, Casadio C (2008). Surgical pleurodesis for Vanderschueren’s stage III primary spontaneous pneumothorax. Eur Respir J.

[162] Ling ZG, Wu YB, Ming MY, Cai SQ, Chen YQ (2015). The effect of pleural abrasion on the treatment of primary spontaneous pneumothorax: a systematic review of randomized controlled trials. PLoS ONE.

[163] Hallifax RJ, Yousuf A, Jones HE, Corcoran JP, Psallidas I, Rahman NM (2017). Effectiveness of chemical pleurodesis in spontaneous pneumothorax recurrence prevention: a systematic review. Thorax.

[164] Gyorik S, Erni S, Studler U, Hodek-Wuerz R, Tamm M, Chhajed PN (2007). Long-term follow-up of thoracoscopic talc pleurodesis for primary spontaneous pneumothorax. Eur Respir J.

[165] Kennedy L, Sahn SA (1994). Talc pleurodesis for the treatment of pneumothorax and pleural effusion. Chest.

[166] Doddoli C, Barlesi F, Fraticelli A, Thomas P, Astoul P, Giudicelli R (2004). Video-assisted thoracoscopic management of recurrent primary spontaneous pneumothorax after prior talc pleurodesis: a feasible, safe and efficient treatment option. Eur J Cardiothorac Surg.

[167] Cardillo G, Facciolo F, Giunti R, Gasparri R, Lopergolo M, Orsetti R (2000). Videothoracoscopic treatment of primary spontaneous pneumothorax: a 6-year experience. Ann Thorac Surg.

[168] Shaikhrezai K, Thompson AI, Parkin C, Stamenkovic S, Walker WS (2011). Video-assisted thoracoscopic surgery management of spontaneous pneumothorax −long-term results. Eur J Cardiothorac Surg.

[169] Asban A, Raza SS, McLeod C, Donahue J, Wei B (2020). Mechanical or chemical and mechanical pleurodesis for spontaneous pneumothorax: what is the most effective approach in preventing recurrence? A systematic review and meta-analysis. Eur J Cardiothorac Surg.

[170] Dearden AS, Sammon PM, Matthew EF (2013). In patients undergoing video-assisted thoracic surgery for pleurodesis in primary spontaneous pneumothorax, how long should chest drains remain in place prior to safe removal and subsequent discharge from hospital?. Interact Cardiovasc Thorac Surg.

[171] Pompili C, Xiume F, Hristova R, Salati M, Refai M, Milton R (2016). Regulated drainage reduces the incidence of recurrence after uniportal video-assisted thoracoscopic bullectomy for primary spontaneous pneumothorax: a propensity case-matched comparison of regulated and unregulated drainagedagger. Eur J Cardiothorac Surg.

[172] Allain PA, Carella M, Agrafiotis AC, Burey J, Assouad J, Hafiani EM (2019). Comparison of several methods for pain management after video-assisted thoracic surgery for pneumothorax: an observational study. BMC Anesthesiol.

[173] Kwon WK, Choi JW, Kang JE, Kang WS, Lim JA, Woo NS (2012). Long thoracic nerve block in video-assisted thoracoscopic wedge resection for pneumothorax. Anaesth Intensive Care.

[174] Baidya DK, Khanna P, Maitra S (2014). Analgesic efficacy and safety of thoracic paravertebral and epidural analgesia for thoracic surgery: a systematic review and meta-analysis. Interact Cardiovasc Thorac Surg.

[175] Junior Ade P, Erdmann TR, Santos TV, Brunharo GM, Filho CT, Losso MJ (2013). Comparison between continuous thoracic epidural and paravertebral blocks for postoperative analgesia in patients undergoing thoracotomy: systematic review. Braz J Anesthesiol.

[176] Scarfe AJ, Schuhmann-Hingel S, Duncan JK, Ma N, Atukorale YN, Cameron AL (2016). Continuous paravertebral block for post-cardiothoracic surgery analgesia: a systematic review and meta-analysis. Eur J Cardiothorac Surg.

[177] Yeung JH, Gates S, Naidu BV, Gao WMJ, SF. (2016). Paravertebral block versus thoracic epidural for patients undergoing thoracotomy. Cochrane Database Syst Rev.

[178] Kosinski S, Fryzlewicz E, Wilkojc M, Cmiel A, Zielinski M (2016). Comparison of continuous epidural block and continuous paravertebral block in postoperative analgaesia after video-assisted thoracoscopic surgery lobectomy: a randomised, non-inferiority trial. Anaesthesiol Intensive Ther.

[179] Khalil AE, Abdallah NM, Bashandy GM, Kaddah TA (2017). Ultrasound-guided serratus anterior plane block versus thoracic epidural analgesia for thoracotomy pain. J Cardiothorac Vasc Anesth.

[180] Fernandez MI, Martin-Ucar AE, Lee HD, West KJ, Wyatt R, Waller DA (2005). Does a thoracic epidural confer any additional benefit following video-assisted thoracoscopic pleurectomy for primary spontaneous pneumothorax?. Eur J Cardiothorac Surg.

[181] Berna P, Quesnel C, Assouad J, Bagan P, Etienne H, Fourdrain A, et al. Enhanced recovery after pulmonary lobectomy. Recommandations formalisées d’experts, SFAR. SFCTCV. 2019. https://sfar.org/rehabilitation-amelioree-apres-lobectomiepulmonaire/. 10.1016/j.accpm.2020.100791 33451912

[182] Lizardo RE, Langness S, Davenport KP, Kling K, Fairbanks T, Bickler SW (2015). Ketorolac does not reduce effectiveness of pleurodesis in pediatric patients with spontaneous pneumothorax. J Pediatr Surg.

[183] Lardinois D, Vogt P, Yang L, Hegyi I, Baslam M, Weder W (2004). Non-steroidal anti-inflammatory drugs decrease the quality of pleurodesis after mechanical pleural abrasion. Eur J Cardiothorac Surg.

[184] Opitz I, Arni S, Oberreiter B, Asmis LM, Vogt P, Rousson V (2013). Perioperative diclofenac application during video-assisted thoracic surgery pleurodesis modulates early inflammatory and fibrinolytic processes in an experimental model. Eur Surg Res.

[185] Ben-Nun A, Golan N, Faibishenko I, Simansky D, Soudack M (2011). Nonsteroidal antiinflammatory medications: efficient and safe treatment following video-assisted pleurodesis for spontaneous pneumothorax. World J Surg.

[186] Dorman RM, Ventro G, Cairo SB, Vali K, Rothstein DH (2018). The use of perioperative ketorolac in the surgical treatment of pediatric spontaneous pneumothorax. J Pediatr Surg.

[187] Hedevang Olesen W, Katballe N, Sindby JE, Titlestad IL, Andersen PE, Ekholm O (2017). Cannabis increased the risk of primary spontaneous pneumothorax in tobacco smokers: a case-control study. Eur J Cardiothorac Surg.

[188] Joobeur S, Cheikh Mhamed S, Mribah H, Skhiri N, Rouetbi N, El Kamel A (2015). Predictive factors of recurrence in spontaneous pneumothorax. Tunis Med.

[189] Hallifax RJ, Goldacre R, Landray MJ, Rahman NM, Goldacre MJ (2018). Trends in the incidence and recurrence of inpatient-treated spontaneous pneumothorax, 19682016. JAMA.

[190] Tulandi T, Sirois C, Sabban H, Cohen A, Murji A, Singh SS (2018). Relationship between catamenial pneumothorax or non-catamenial pneumothorax and endometriosis. J Minim Invasive Gynecol.

[191] Gil Y, Tulandi T (2020). Diagnosis and treatment of catamenial pneumothorax: a systematic review. J Minim Invasive Gynecol.

[192] Ruppert AM, Sroussi D, Khallil A, Giot M, Assouad J, Cadranel J (2020). Detection of secondary causes of spontaneous pneumothorax: comparison between computed tomography and chest X-ray. Diagn Interv Imaging.

[193] Johannesma PC, Reinhard R, Kon Y, Sriram JD, Smit HJ, van Moorselaar RJ (2015). Prevalence of Birt-Hogg-Dube syndrome in patients with apparently primary spontaneous pneumothorax. Eur Respir J.

[194] Villela MA, Dunworth S, Harlan NP, Moon RE (2018). Can my patient dive after a first episode of primary spontaneous pneumothorax? A systematic review of the literature. Undersea Hyperb Med.

[195] Martinez-Ramos D, Angel-Yepes V, Escrig-Sos J, Miralles-Tena JM, Salvador-Sanchis JL (2007). Usefulness of computed tomography in determining risk of recurrence after a first episode of primary spontaneous pneumothorax: therapeutic implications. Arch Bronconeumol.

[196] Laituri CA, Valusek PA, Rivard DC, Garey CL, Ostlie DJ, Snyder CL (2011). The utility of computed tomography in the management of patients with spontaneous pneumothorax. J Pediatr Surg.

[197] Almajid FM, Aljehani YM, Alabkary S, Alsaif HS (2019). The accuracy of computed tomography in detecting surgically resectable blebs or bullae in primary spontaneous pneumothorax. Radiol Med.

[198] Kim JT, Oh TY, Chang WH, Kong JH, Baek KS, Lee WJ (2014). Natural course of spontaneous pneumothorax without bullae or blebs under high-resolution computed tomography. Thorac Cardiovasc Surg.

[199] Beregi JP, Greffier J (2019). Low and ultra-low dose radiation in CT: opportunities and limitations. Diagn Interv Imaging.

[200] Miller AR, Jackson D, Hui C, Deshpande S, Kuo E, Hamilton GS (2019). Lung nodules are reliably detectable on ultra-low-dose CT utilising model-based iterative reconstruction with radiation equivalent to plain radiography. Clin Radiol.

[201] Akcam TI, Kavurmaci O, Ergonul AG, Aydin S, Turhan K, Cakan A (2018). Analysis of the patients with simultaneous bilateral spontaneous pneumothorax. Clin Respir J.

[202] Lippert HL, Lund O, Blegvad S, Larsen HV (1991). Independent risk factors for cumulative recurrence rate after first spontaneous pneumothorax. Eur Respir J.

[203] Bock K, Lohse Z, Madsen PH, Hilberg O (2018). Birt-Hogg-Dube syndrome: spontaneous pneumothorax as a first symptom. BMJ Case Rep.

[204] Gupta N, Langenderfer D, McCormack FX, Schauer DP, Eckman MH (2017). Chest computed tomographic image screening for cystic lung diseases in patients with spontaneous pneumothorax is cost effective. Ann Am Thorac Soc.

[205] Duchateau FX, Legrand JM, Verner L, Brady WJ (2013). Commercial aircraft repatriation of patients with pneumothorax. Air Med J.

[206] Ahmedzai S, Balfour-Lynn IM, Bewick T, Buchdahl R, Coker RK, Cummin AR (2011). Managing passengers with stable respiratory disease planning air travel: British thoracic society recommendations. Thorax.

[207] Lynch JH, Deaton TG (2014). Barotrauma with extreme pressures in sport: from scuba to skydiving. Curr Sports Med Rep.

[208] Fer C, Guiavarch M, Edouard P (2021). Epidemiology of skydiving-related deaths and injuries: a 10-years prospective study of 6.2 million jumps between 2010 and 2019 in France. J Sci Med Sport.

[209] Christey GR (2005). Serious parasport injuries in Auckland New Zealand. Emerg Med Australas.

[210] Russi EW (1998). Diving and the risk of barotrauma. Thorax.

[211] de Bakker HM, Tijsterman M, de Bakker-Teunissen OJG, Soerdjbalie-Maikoe V, van Hulst RA, de Bakker BS (2020). Prevalence of pulmonary bullae and blebs in postmortem CT imaging with potential implications for diving medicine. Chest.

[212] Tetzlaff K, Reuter M, Leplow B, Heller M, Bettinghausen E (1997). Risk factors for pulmonary barotrauma in divers. Chest.

[213] Lippmann J, Mc DTD, Stevenson C, Williams J, Mitchell SJ (2017). Diving with pre-existing medical conditions. Diving Hyperb Med.

[214] Cattoni M, Rotolo N, Mastromarino MG, Cardillo G, Nosotti M, Mendogni P (2020). Analysis of pneumothorax recurrence risk factors in 843 patients who underwent videothoracoscopy for primary spontaneous pneumothorax: results of a multicentric study. Interact Cardiovasc Thorac Surg.

[215] Germonpre P, Van Renterghem E, Dechamps N, Onghena T, Van Aken J (2020). Recurrence of spontaneous pneumothorax six years after VATS pleurectomy: evidence for formation of neopleura. J Cardiothorac Surg.

[216] Bense L, Wiman LG, Hedenstierna G (1987). Onset of symptoms in spontaneous pneumothorax: correlations to physical activity. Eur J Respir Dis.

[217] Dejene S, Ahmed F, Jack K, Anthony A (2013). Pneumothorax, music and balloons: a case series. Ann Thorac Med.

