# Supplementary material for: SPLF/SMFU/SRLF/SFAR/SFCTCV Guidelines for the management of patients with primary spontaneous pneumothorax
Source: Ann Intensive Care. 2023 Sep 19;13:88. doi: 10.1186/s13613-023-01181-2 (PMC10509123; doi:10.1186/s13613-023-01181-2)
Supplement: Supplementary file 1 — Additional file 1: Appendix S1. Table presenting the bibliographic search equations in PubMed. Appendix S2. Different methods for assessing pneumothorax size. Figure S1. Schematic representation of the method for assessing pneumothorax size: Rhea’s method. Figure S2. Schematic representation of the method for assessing pneumothorax size: Collins’ method. Figure S3. Schematic representation of the method for assessing pneumothorax size: Light’s method. Appendix S3. High-risk occupations requiring adjustments with regard to surgical indications [file 13613_2023_1181_MOESM1_ESM.docx]

**APPENDICES**

**Appendix 1: Table presenting the bibliographic search equations in PubMed**

| Question | | | | | Equation |
| --- | --- | --- | --- | --- | --- |
| **P** | **P1** | **I** | **C** | **O** |  |
| suspected PSP |  | CT scan | *vs.* CXR = XRss = chest X-ray |  | ("(("pneumothorax" AND ( "diagnosis" OR "suspicion") AND ("radiography" AND "ultrasonography")) ))) OR (pneumothorax/diagnosis[MeSH Major Topic]) AND Radiography, Thoracic[MeSH Terms] AND Ultrasonography[MeSH Terms])  /methods"[Mesh] AND "pneumothorax"[MeSH]) OR ("pneumothorax"[All Fields] AND ("diagnosis"[All Fields] OR "suspicion"[All Fields]) AND ("radiography"[All Fields] AND ("CT X Ray"[All Fields] OR "tomodensitometry"[All Fields]))) |
| suspected PSP |  | CT scan | *vs.* chest/pleural ultrasound |  | (("pneumothorax" AND ( "diagnosis" OR "suspicion") AND ("ultrasonography" AND ("CT X Ray" OR "tomodensitometry") ))) OR (("pneumothorax/diagnosis"[MAJR] AND "tomography, x-ray computed"[MeSH Terms]) AND "ultrasonography"[MeSH Terms]) |
| suspected PSP |  | Ultrasound | *vs.* XR |  | (("pneumothorax" AND ( "diagnosis" OR "suspicion") AND ("radiography" AND "ultrasonography")) ))) OR (pneumothorax/diagnosis[MeSH Major Topic]) AND Radiography, Thoracic[MeSH Terms] AND Ultrasonography[MeSH Terms]) |
| what is the best method to assess PSP size on XR |  |  |  |  | (("Radiography, Thoracic/methods"[Mesh] AND "pneumothorax"[MeSH]) OR (("Radiography" AND "method" AND "evaluation") AND "pneumothorax") |
| PSP with respiratory distress | Extreme emergency = on demand needle aspiration |  |  |  | (("respiratory distress syndrome, adult"[MeSH Terms] AND "pneumothorax"[Majr]) AND (("critical illness"[MeSH Terms] OR "critical care"[MeSH Terms]) OR "emergencies"[MeSH Terms]) |
| PSP with respiratory distress | Non-extreme emergency, with clinical severity criteria |  |  |  | ("respiratory distress syndrome, adult"[MeSH Terms] AND "pneumothorax"[Majr]) |
| PSP with respiratory distress | Non-extreme emergency, with clinical severity criteria | drainage duration / | hospitalisation |  | (("respiratory distress syndrome, adult"[MeSH Terms] AND "pneumothorax"[Majr]) AND ( "drainage"[MeSH Terms] OR "hospitalization"[MeSH Terms])) |
| PSP with respiratory distress | Non-extreme emergency, with clinical severity criteria |  |  | complications | ("respiratory distress syndrome, adult"[MeSH Terms] AND "pneumothorax/complications"[Majr]) |
| PSP with respiratory distress | Non-extreme emergency, with clinical severity criteria |  |  | survival | "respiratory distress syndrome, adult"[MeSH Terms] AND "pneumothorax"[Majr]) AND ("survival rate"[MeSH Terms] OR (survival)) |
| PSP with respiratory distress | Non-extreme emergency, with clinical severity criteria |  |  | recurrence | ((("respiratory distress syndrome, adult"[MeSH Terms] AND "pneumothorax"[Majr]))) AND "recurrence"[MeSH Terms] |
| PSP (without distress) PSP (large without severity criteria) |  | drainage | *vs.* needle aspiration |  | ((("pneumothorax"[All Fields] AND "primary spontaneous"[All Fields]) OR "PSP"[All Fields]) AND 2018/1/1:3000/12/31[Date - Publication]) OR "pneumothorax"[MeSH Major Topic])) AND (((("thoracentesis"[MeSH Terms] OR "aspiration"[All Fields]) OR "exsufflation"[All Fields]) OR "drain"[All Fields]) OR "drainage/methods"[MeSH Terms]) |
| PSP (without distress) PSP (large without severity criteria) |  | drainage | *vs.* conservative |  | (((("pneumothorax"[All Fields] AND "primary spontaneous"[All Fields]) OR "psp"[All Fields]) AND 2018/1/1:3000/12/31[Date - Publication]) OR "pneumothorax"[MeSH Major Topic])) AND ("conservative treatment"[MeSH Terms] OR ("conservative"[All Fields] AND 2018/1/1:3000/12/31[Date - Publication])) |
| PSP (without distress) PSP (large without severity criteria) |  | drainage | *vs.* surgery |  | ((((("pneumothorax"[All Fields] AND "primary spontaneous"[All Fields]) OR "psp"[All Fields]) AND 2018/1/1:3000/12/31[Date - Publication]) OR "pneumothorax"[MeSH Major Topic])) AND (("thoracic surgery"[All Fields] AND 2019/1/1:3000/12/31[Date - Publication]) OR "Thoracic Surgical Procedures"[MeSH Terms]))) AND ("aspiration"[All Fields] OR "drain"[All Fields] OR "drainage/methods"[MeSH Terms])) |
| In PSP (**small** without severity criteria) |  | drainage duration / | hospitalisation |  | ((("hospitalization"[MeSH Terms] OR ("hospitalization"[All Fields] AND 2018/1/1:3000/12/31[Date - Publication])) AND (((("pneumothorax"[All Fields] AND "primary spontaneous"[All Fields]) OR "PSP"[All Fields]) AND ("2018"[PDAT] : "3000"[PDAT])) OR "pneumothorax"[MeSH Major Topic])) AND (mild OR "low grade") |
| In PSP (**small** without severity criteria) |  | drainage duration / | hospitalisation | complications | (((("hospitalization"[MeSH Terms] OR ("hospitalization"[All Fields] AND 2018/1/1:3000/12/31[Date - Publication])) AND (((("pneumothorax"[All Fields] AND "primary spontaneous"[All Fields]) OR "PSP"[All Fields]) AND ("2018"[PDAT] : "3000"[PDAT])) OR "pneumothorax"[MeSH Major Topic])) AND (Complications[MeSH Subheading] OR "complication"[All Fields]) |
| In PSP (**small** without severity criteria) |  | drainage duration / | hospitalisation | survival | ((((("hospitalization"[MeSH Terms] OR ("hospitalization"[All Fields] AND 2018/1/1:3000/12/31[Date - Publication])) AND (((("pneumothorax"[All Fields] AND "primary spontaneous"[All Fields]) OR "PSP"[All Fields]) AND ("2018"[PDAT] : "3000"[PDAT])) OR "pneumothorax"[MeSH Major Topic])) ) AND ("survival"[MeSH Major Topic] OR "survival"[All Fields]) |
| In PSP (**small** without severity criteria) |  | drainage duration / | hospitalisation | recurrence | ((((("hospitalization"[MeSH Terms] OR ("hospitalization"[All Fields] AND 2018/1/1:3000/12/31[Date - Publication])) AND (((("pneumothorax"[All Fields] AND "primary spontaneous"[All Fields]) OR "PSP"[All Fields]) AND ("2018"[PDAT] : "3000"[PDAT])) OR "pneumothorax"[MeSH Major Topic])) ) AND ("recurrence"[MeSH Major Topic] OR "recurrence"[All Fields]) |
| In PSP (**small** without severity criteria) |  | hospitalisation for monitoring | *vs.* outpatient | complications | ((("primary spontaneous pneumothorax"[All Fields] OR "PSP"[All Fields]) AND ("2018"[PDAT] : "3000"[PDAT])) OR "pneumothorax"[MeSH Terms]) AND ((aftercare[MeSH Terms]) OR ("follow-up"[All Fields] OR "aftercare"[All Fields] AND 2018/1/1:3000/12/31[Date - Publication])) AND ((("outpatients"[MeSH Terms] OR "ambulatory care"[MeSH Terms] OR "hospitalization"[MeSH Terms] OR "inpatients"[MeSH Terms]) OR (("outpatients"[All Fields] OR "ambulatory"[All Fields] OR "hospitalization"[All Fields] OR "inpatients"[All Fields] ) AND 2018/1/1:3000/12/31[Date - Publication])) |
| PSP, one of these complications: | Bilateral, hydroaeric level, adhesion | X-ray | *vs.* absence | complications | ("pneumothorax/complications"[MeSH Major Topic] OR (("bilateral" OR "compressive" OR "hemorragic") AND "primary spontaneous pneumothorax") OR (("hemopneumothorax"[MeSH Terms] OR "hydropneumothorax"[MeSH Terms]) AND "primary spontaneous" ) AND (("radiography") OR "Radiography, Thoracic"[MeSH Terms]) |
|  |  |  |  | recurrence | (("recurrence"[MeSH Major Topic] OR "recurrence"[All Fields])) AND (("pneumothorax/complications"[MeSH Major Topic] OR (("bilateral" OR "compressive" OR "hemorragic") AND "primary spontaneous pneumothorax") OR (("hemopneumothorax"[MeSH Terms] OR "hydropneumothorax"[MeSH Terms]) AND "primary spontaneous" ) AND (("radiography") OR "Radiography, Thoracic"[MeSH Terms])) |
| PSP |  | Drainage / chest tube size | needle aspiration |  | (("primary spontaneous pneumothorax" AND 2018/1/1:3000/12/31[Date - Publication]) OR "pneumothorax"[MeSH Major Topic]) AND (("thoracentesis"[MeSH Terms] OR "aspiration"[All Fields] OR "drain"[All Fields] OR "drainage/methods"[MeSH Terms]) AND ("diameter")) |
| PSP | drained patients (= SOC) | Axillary drainage location | Anterior drainage location |  | (((("pneumothorax"[All Fields] AND "primary spontaneous"[All Fields]) AND 2018/1/1:3000/12/31[Date - Publication]) OR "pneumothorax"[MeSH Major Topic]) AND "thoracentesis/methods"[MeSH Terms]) OR (("needles"[MeSH Terms] AND ((("pneumothorax"[All Fields] AND "primary spontaneous"[All Fields]) AND 2018/1/1:3000/12/31[Date - Publication]) OR "pneumothorax"[MeSH Major Topic])) AND ((("thoracic"[All Fields] OR "chest"[All Fields]) AND ("drain"[All Fields] OR "aspiration"[All Fields])) OR "thoracentesis/methods"[MeSH Terms])) |
| PSP | drained patients (= SOC) | aspiration | *vs.* no | edema a vacuo | (("thoracentesis"[MeSH Terms] OR "drainage"[MeSH Terms]) AND ((((("edema"[MeSH Terms] OR "edema"[All Fields]) OR "edemas"[All Fields]) OR "oedemas"[All Fields]) OR "oedema"[All Fields]) OR "pulmonary edema"[MeSH Terms])) AND ((("pneumothorax"[All Fields] AND "primary spontaneous"[All Fields]) AND 2018/1/1:3000/12/31[Date - Publication]) OR "pneumothorax"[MeSH Terms]) |
| PSP | drained patients (= SOC) |  |  | pain | ("pain, procedural"[MeSH Terms]) AND (("pneumothorax"[All Fields] AND "primary spontaneous"[All Fields] AND 2018/1/1:3000/12/31[Date - Publication]) OR "pneumothorax"[MeSH Terms])) |
| PSP | drained patients (= SOC) | Clamping | *vs.* no |  | (("thoracentesis"[MeSH Terms] OR "drainage/methods"[MeSH Terms] OR "needles"[MeSH Terms]) AND (("pneumothorax"[All Fields] AND "primary spontaneous"[All Fields] AND 2018/1/1:3000/12/31[Date - Publication]) OR "pneumothorax"[MeSH Terms]))) AND (("constriction"[MeSH Terms]) OR ((clamp OR clamping) AND 2018/1/1:3000/12/31[Date - Publication])) |
| PSP | drained patients (= SOC) | Drainage duration: minimum drainage duration before removal |  |  | ((duration)) AND ((((("pneumothorax"[All Fields] AND "primary spontaneous"[All Fields]) AND 2018/1/1:3000/12/31[Date - Publication]) OR "pneumothorax"[MeSH Major Topic]) AND "thoracentesis/methods"[MeSH Terms]) OR (("needles"[MeSH Terms] AND ((("pneumothorax"[All Fields] AND "primary spontaneous"[All Fields]) AND 2018/1/1:3000/12/31[Date - Publication]) OR "pneumothorax"[MeSH Major Topic])) AND ((("thoracic"[All Fields] OR "chest"[All Fields]) AND ("drain"[All Fields] OR "aspiration"[All Fields])) OR "thoracentesis/methods"[MeSH Terms]))) Sort by: Most Recent |
| PSP | drained patients (= SOC) |  | Oxygen therapy |  | (("oxygen inhalation therapy"[MeSH Terms] OR "Oxygen Inhalation"[All Fields])) AND (("pneumothorax"[All Fields] AND "primary spontaneous"[All Fields] AND 2018/1/1:3000/12/31[Date - Publication]) OR "pneumothorax"[MeSH Terms])) NOT (conservative)) |
| PSP | drained patients (= SOC) |  | Oxygen therapy | reexpansion duration | (("oxygen inhalation therapy"[MeSH Terms] OR "Oxygen Inhalation"[All Fields]) AND ((("pneumothorax"[All Fields] AND "primary spontaneous"[All Fields]) AND 2018/1/1:3000/12/31[Date - Publication]) OR "pneumothorax"[MeSH Terms])) AND ("reexpansion"[All Fields] OR "re-expansion"[All Fields]) |
| PSP | drained patients (= SOC) |  | Oxygen therapy | complications | ("oxygen inhalation therapy"[MeSH Major Topic] AND ((("pneumothorax"[All Fields] AND "primary spontaneous"[All Fields]) AND 2018/1/1:3000/12/31[Date - Publication]) OR "pneumothorax"[MeSH Terms])) AND ("complications"[MeSH Subheading] OR "complications"[All Fields]) |
| PSP | drained patients (= SOC) |  | Oxygen therapy | survival | ("oxygen inhalation therapy"[MeSH Major Topic]) AND (("pneumothorax"[All Fields] AND "primary spontaneous"[All Fields] AND 2018/1/1:3000/12/31[Date - Publication]) OR "pneumothorax"[MeSH Terms])) AND (survival) |
| PSP | drained patients (= SOC) |  | Oxygen therapy | recurrence | ("oxygen inhalation therapy"[MeSH Major Topic] OR "oxygen inhalation") AND (("pneumothorax"[All Fields] AND "primary spontaneous"[All Fields] AND 2018/1/1:3000/12/31[Date - Publication]) OR "pneumothorax"[MeSH Terms])) AND (recurrence) |
| PSP | drained patients (= SOC) |  |  | Monitoring | ((("pneumothorax"[All Fields] AND "primary spontaneous"[All Fields] AND 2018/1/1:3000/12/31[Date - Publication]) OR "pneumothorax"[MeSH Terms]) AND ((aftercare[MeSH Terms] OR "follow-up"[All Fields] OR "aftercare"[All Fields] OR "patient monitoring"[All Fields]) AND ("thoracentesis"[MeSH Terms] OR "drainage"[MeSH Terms] OR "needles"[MeSH Terms] OR "aspiration"[All Fields] OR "exsufflation"[All Fields] OR "drain"[All Fields])))) |
| PSP |  | drained patients (= SOC) | needle aspiration | Antalgia | ("pain/drug therapy"[MeSH Terms] OR "Anesthesia and Analgesia"[MeSH Terms] OR (analgesia) OR (pain medication)) AND (((("pneumothorax"[All Fields] AND "primary spontaneous"[All Fields]) OR "pneumothorax"[MeSH Major Topic]) AND ("thoracentesis"[MeSH Terms] OR "drainage"[MeSH Terms] OR "needles"[MeSH Terms] OR "aspiration"[All Fields] OR "exsufflation"[All Fields] OR "drain"[All Fields])))) |
| In conservatively-treated PSP |  | monitoring interval H |  |  | (aftercare[MeSH Terms] OR "follow-up"[All Fields] OR "aftercare"[All Fields] OR "patient monitoring"[All Fields]) AND (pneumothorax) AND ("conservative treatment"[MeSH Terms] OR (conservative)) |
| In conservatively-treated PSP |  | Strict bed rest |  |  | (bed rest) AND ("conservative treatment"[MeSH Terms] OR (conservative)) AND (pneumothorax) |
| PSP | medical transfer by AIRPLANE | drainage | *vs.* no |  | (("patient transfer"[MeSH Terms] OR "patient transfer") AND (air)) AND (pneumothorax) |

**Appendix 2: Different methods for assessing pneumothorax size.**

The size of a pneumothorax may be estimated on chest X-ray performed in a standing position as follows:

**For the CEP (French College of Respiratory Teachers)** (1) **and for the BTS (British Thoracic Society)** (2)**:** A large pneumothorax is arbitrarily defined as the presence of a visible rim along the entire axillary line, ≥2 cm between the lung margin and the chest wall at the hilum level.

1.
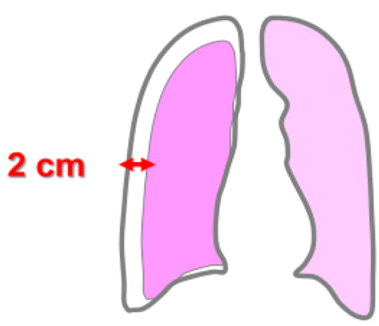


**For the BSP (Belgian Society of Pulmonology)** (3) **and the SEPAR (Spanish Society of Pulmonology and Thoracic Surgery)** (4)**:** a displacement of the pleural line along the entire lateral chest wall.

**For the ACCP (American College of Chest Physicians)** (5)**:** interpleural distance >3 cm at the apex


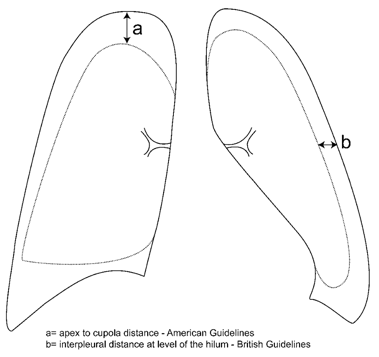


a = distance between the apex and the pleural line - ACCP guidelines

b = interpleural distance at the hilum - BTS guidelines

1. **Rhea's method (6):** The percentage of displacement of the pleural line represents the mean of the distance of the displacement at 3 points:

**% Rhea= 5 + 1(35/12)(A+B+C)**

| 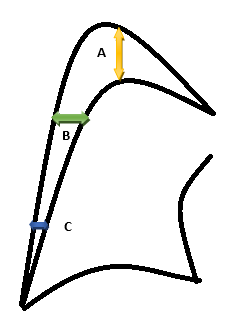 | A = the maximum distance at the apex  B = the median distance from the upper half of the lung  C = the median distance from the lower half of the lung |
| --- | --- |

**Figure 1: Schematic representation of the method for assessing pneumothorax size: Rhea's method**

The Rhea's method is more accurate in estimating small pneumothoraces but underestimates the size of large pneumothoraces.

1. **Collins’ method (7)**: Calculation of the percentage of displacement of the pleural line:

**% Collins = 4.2 + 4.7x(A+B+C)**

| 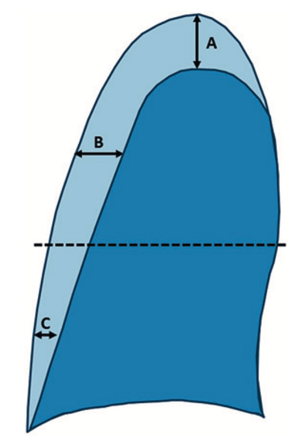 | A = the maximum distance at the apex  B = the median distance from the upper half of the lung  C = the median distance from the lower half of the lung |
| --- | --- |

**Figure 2. Schematic representation of the method for assessing pneumothorax size: Collins’ method**

The Collins' method appears to be more accurate, especially in the assessment of large pneumothoraces. A pneumothorax is considered large when the sum of the measured values is ≥4 cm.

1. **Light’s index** (8): Measurement of pneumothorax size:

**% Light = ( 1 – b3 / a3 ) x 100**

| 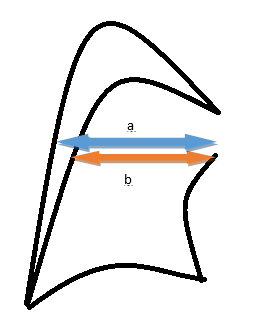 | a = diameter of the hemithorax at the hilum  b = diameter of the lung at the hilum |
| --- | --- |

**Figure 3: Schematic representation of the method for assessing pneumothorax size: Light’s method.**

A pneumothorax is considered small if the % is <20%, medium if it is equal to 20-50% and large if it is >50%.

This index seems to be less accurate (9). It underestimates the pneumothorax size by 7.3% on average compared to the calculation made on CT images, derived from the Collins' method.

A study comparing the pneumothorax size classifications used by different guidelines has shown a poor agreement between them, and there is no current evidence to support the treatment decision based on pneumothorax size alone (1).

**Appendix 3: High-risk occupations requiring adjustments with regard to surgical indications**

- 1. **Soldiers**
     1. **General case**

In general, a first episode of pneumothorax results in an inability to participation in field operations, short-term overseas missions for a period of three years, provided that normal physical examination, a chest CT-scan and PFT are obtained. This period may be reduced to 3 months after pleurodesis, provided that normal physical examination, a chest CT-scan and PFT are obtained.

- - 1. **Airborne troops**

A history of pneumothorax results in definite inability for military skydiving. This inability may be reduced to 3 months after pleurodesis, provided that the above-mentioned conditions are met.

- - 1. **Flight attendants**
       1. *Fighter pilots*

The limitation is that of the A standard, required for ejection seat capability. A history of pneumothorax results in definite incapability for ejection seat. This incapability may be lifted 3 months after pleurodesis is performed, provided that the above-mentioned conditions are met and stress tests are performed (centrifuge, hypobaric chamber). In case of proven contralateral bullous dystrophy, pleural resection and pleurodesis should be performed.

- - - 1. *Other military pilots*

In a co-pilot, a first episode of pneumothorax results in a 6-month inability provided that the above-mentioned conditions are met and stress tests are performed (centrifuge, hypobaric chamber). This inability may be reduced to 3 months after pleurodesis, provided that the above-mentioned conditions are met.

- - - 1. *Other crew members*

A first episode of pneumothorax results in a 6-week inability provided that the above-mentioned conditions are met.

- - 1. **Underwater occupations**
       1. *Scuba divers*

A history of pneumothorax results in a definite inability for scuba diving, including after pleurodesis.

- - - 1. *Submariners*

A history of pneumothorax results in definite inability for underwater navigation. This incapability may be reduced to 3 months after pleurodesis, provided that the above-mentioned conditions are met.

- 1. **Civil aviation aircrew**
     1. **Airline pilots**

A first episode of pneumothorax results in a 1-year inability provided that the above-mentioned conditions are met. This period may be reduced to 3 months after pleurodesis is performed, provided that the above-mentioned conditions are met, or to 6 weeks, provided that the above-mentioned conditions are met and that there is an Operational Multi-pilot Limitation (OML).

- - 1. **Other crew members**

A first episode of pneumothorax results in a 6-week incapability provided that the above-mentioned conditions are met.

- 1. **High mountain professionals**

The high mountain corresponds to an altitude greater than 8,000 feet (2,448 m) exposing to the risk of acute mountain sickness, and high-altitude cerebral and pulmonary edema.

Given the rate of recurrence, the risk of hypoxia and the difficulties of access, the group proposes to perform pleurodesis from the first episode of spontaneous pneumothorax.

- 1. **Workers in areas with limited health coverage**

Given the rate of recurrence, depending on the difficulties in accessing care, the group proposes to perform pleurodesis from the first episode of spontaneous pneumothorax.
